# Polyploid *Nicotiana* section *Suaveolentes* originated by hybridization of two ancestral *Nicotiana* clades

**DOI:** 10.3389/fpls.2023.999887

**Published:** 2023-05-08

**Authors:** Lucio D’Andrea, Nicolas Sierro, Sonia Ouadi, Tomas Hasing, Elijah Rinaldi, Nikolai V. Ivanov, Aureliano Bombarely

**Affiliations:** ^1^ Department of Bioscience, Universita degli Studi di Milano, Milan, Italy; ^2^ PMI R&D, Philip Morris Products S.A., Quai Jeanrenaud 5, Neuchâtel, Switzerland; ^3^ Elo Life Systems, Durham, NC, United States; ^4^ School of Plant and Environmental Sciences, Virginia Tech, Blacksburg, VA, United States; ^5^ Instituto de Biologia Molecular y Celular de Plantas (IBMCP) (CSIC-UPV), Valencia, Spain

**Keywords:** *Nicotiana*, polyploidy, phylogenomic, phylogenetic dating, plastidgenome recombination

## Abstract

**Introduction:**

*Nicotiana* section *Suaveolentes* is an almost all-Australian clade of allopolyploid tobacco species that emerged through hybridization between diploid relatives of the genus. In this study, we aimed to assess the phylogenetic relationship of the *Suaveolentes* section with several *Nicotiana* diploid species based on both plastidial and nuclear genes.

**Methods:**

The *Nicotiana* plastome-based phylogenetic analysis representing 47 newly re-built plastid genomes suggested that an ancestor of *N*. section *Noctiflorae* is the most likely maternal donor of the *Suaveolentes* clade. Nevertheless, we found clear evidence of plastid recombination with an ancestor from the *Sylvestres* clade. We analyzed 411 maximum likelihood-based phylogenetic trees from a set of conserved nuclear diploid single copy gene families following an approach that assessed the genomic origin of each homeolog.

**Results:**

We found that *Nicotiana* section *Suaveolentes* is monophyletic with contributions from the sections *Alatae*, *Sylvestres*, *Petunioides* and *Noctiflorae*. The dating of the divergence between these sections indicates that the *Suaveolentes* hybridization predates the split between *Alatae/Sylvestres*, and *Noctiflorae/Petunioides*.

**Discussion:**

We propose that *Nicotiana* section *Suaveolentes* arose from the hybridization of two ancestral species from which the *Noctiflorae/Petunioides* and *Alatae/Sylvestres* sections are derived, with *Noctiflorae* the maternal parent. This study is a good example in which the use of genome wide data provided additional evidence about the origin of a complex polyploid clade.

## 1 Introduction

*Nicotian*a, an herbaceous plant genus, belongs to the Solanaceae family. The genus includes 90 naturally occurring species divided into 13 sections, of which five contain allopolyploids formed by interspecific hybridization between diploids (Knapp et al., 2004; Bally et al., 2021; Chase et al., 2021; Augsten et al., 2022; Santilli et al., 2022). The species of the *Nicotiana* genus vary greatly in genome size and chromosome number based on their sections (Leitch et al., 2008). For example, although the haploid genome size (1C) of diploid *N. obtusifolia* (2n = 2x = 22) is 1.51 Gb (Leitch et al., 2008), that of the allopolyploid *N. tabacum* (2n = 4x = 48) is approximately 4.5 Gb. *Suaveolentes* is one of the oldest polyploid sections of the *Nicotiana* genus and includes 38 species (Chase et al., 2021), such as the well-studied *Nicotiana benthamiana* (Cauz-Santos et al., 2022). Molecular dating and phylogenetic analysis indicate that the *Suaveolentes* clade arose from a single hybridization event (*circa* 6 Mya) (Chase et al., 2003; Chase et al., 2018). Biogeographic surveys have revealed that the majority of *Suaveolentes* species exist in Australia, with a few present in other areas such as Namibia, New Caledonia, and a group of isolated islands in the Pacific Ocean (Ladiges et al., 2011). Australian *Nicotiana* species are widely distributed across the country and in several bioregions. This extensive distribution of this section has been attributed to a single allopolyploidization event followed by a rapid adaptation and a high speciation rate rather than features coming from its ancestors (Leitch et al., 2008; Ladiges et al., 2011; Clarkson et al., 2017; Chase et al., 2018; Dodsworth et al., 2020)

Several efforts have been made to elucidate the origin of the section *Suaveolentes* (Goodspeed, 1954; Clarkson et al., 2010; Kelly et al., 2013; Schiavinato et al., 2020; Schiavinato et al., 2021). The first attempts, based on morphological and/or genetic characterization, revealed that this section was derived from ancestors of the present-day sections *Alatae* and *Sylvestres* and that of the sections *Petunioides* and *Noctiflorae* (Goodspeed, 1954; Clarkson et al., 2010; Kelly et al., 2013). Recently, two articles describing phylogenetic analyses focused on the origin of *N. benthamiana* identified the sections *Noctiflorae* and *Sylvestres* as genome donors (Schiavinato et al., 2020; Schiavinato et al., 2021). Plastome phylogenetic analysis was conducted to verify the maternal contribution of the *Suaveolentes* section (Chase et al., 2003; Clarkson et al., 2004; Kelly et al., 2013; Schiavinato et al., 2020), although independent studies have provided conflicting evidence. Most of the published studies proposed section *Noctiflorae*/*Petunioides* as the most likely maternal donor (Chase et al., 2003; Clarkson et al., 2004; Kelly et al., 2013; Schiavinato et al., 2020), with one exception in which section *Sylvestres* is suggested as the most likely maternal ancestor using a different set of genetic markers (Clarkson et al., 2010). This inconsistency could have been due to different processes, such as incomplete lineage sorting (ILS) and/or hybridization (Kelly et al., 2013). The same study, involving maximum parsimony analysis and phylogenetic supernetworks, proposed that the recurrent gene tree discordances are more likely due to hybridization (and/or introgression) between the progenitors. Thus, Kelly et al. (Kelly et al., 2013) proposed that section *Suaveolentes* arose from a single allopolyploidization event involving an ancestral member of section *Sylvestres* and a hypothetical diploid species that contains alleles from sections *Petunioides* and *Noctiflorae*.

The complex genomic evolution of the clade, the limited number of evaluated loci per taxa, and the total number of species analyzed are likely responsible for these contradictions. Hence, to elucidate the origins of the *Suaveolentes* section, we performed the following analyses: (1) phylogenetic analysis based on 54 whole-plastome sequences representing all *Nicotiana* sections; (2) high-throughput phylogenetic analysis based on nuclear gene families that distinguish the origins of each homoelog gene in four polyploids species of the *Suaveolentes* section; and (3) dating of the ancestral speciation event for the sections *Alatae, Noctiflorae, Petunioides, Sylvestres* and *Suaveolentes* by two independent approaches.

Whole-plastome-based maximum-likelihood (ML) and Bayesian inference analyses (BI) assigned sections *Noctiflorae* and *Sylvestres* as respective maternal donors. This contradictory result is due to a recombination event in which the inverted repeats were derived from *Sylvestres* and the rest of the plastid genome of *Noctiflorae*. Nuclear gene-based high-throughput analysis revealed the genetic contribution of each present-day diploid species in the *Suaveolentes* section. Section *Suaveolentes* possesses nuclear gene families from sections *Alatae*, *Noctiflorae*, *Petunioides*, and, to a lesser extent, *Sylvestres*. The dating of the different diversification events suggests that the *Suaveolentes* section arose from the hybridization of common ancestors of section *Alatae* and *Sylvestres*, as one parent and section *Noctiflorae* and *Petunioides* as the other parent.

## 2 Material and methods

### 2.1 Plant material

Seeds for the whole-genome sequencing of 18 *Nicotiana* species as well as other diploid species (**Supplementary Table S1**) were obtained from the Agricultural Research System (ARS) Germplasm Resources Information Network (GRIN, Beltsville, MD, USA). After sowing the seeds in a standard soil, the plants were grown in a greenhouse at a 16-h light:8-h dark cycle. Leaf samples were collected from adult plants, ground in liquid nitrogen, lyophilized, and stored at room temperature until DNA extraction.

### 2.2 DNA extraction, library preparation, and sequencing

DNA for whole-genome sequencing of the 18 *Nicotiana* species (**Supplementary Table S2**) was extracted from aerial parts of one single plant of each species using a Qiagen DNeasy Plant Maxi Kit (Qiagen, Hilden, Germany), and the quality and quantity of the extracted DNA were verified using a Bioanalyzer (Agilent Technologies, Santa Clara, CA, USA). Following fragmentation using a Covaris E220 Focused-ultrasonicator (LGC Genomics, Berlin, Germany), paired-end sequencing libraries were prepared using an Illumina TruSeq DNA Sample Preparation Kit v2 (Illumina, San Diego, CA, USA). All libraries were sequenced on an Illumina HiSeq-2500 sequencer (Illumina) using v3 chemistry and flow cells with runs of 2 × 100 bases. Each accession was sequenced with a minimum genome coverage of 40X according to the genome size previously estimated by flow cytometry (**Supplementary Table S1**). Except for *N. hesperis*, all the samples reached the sequencing goal (**Supplementary Table S2**).

### 2.3 NCBI Data mining

Illumina pair end reads were downloaded from the NCBI SRA database as follows: 1) navigating to “Nicotiana” in the Taxonomy NCBI database; 2) selecting the “SRA Experiments” box and clicking on the display button; 3) selecting the number of SRA experiments and clicking on “Send results to Run selector”; 4) filtering SRA runs by Platform = “Illumina”, Assay Type = “wgs” and “rna-seq” and Library Layout = “paired”; and 5) selecting the datasets and clicking on “Run Info Table.” Once the table was downloaded, the lines containing the species that were sequenced at the time of data mining were filtered out (“*N. tabacum*,” “*N. benthamiana*,” “*N. attenuata*,” “*N. sylvestris*,” and “*N. tomentosiformis*”). Then, three columns were selected: Run, Assay Type, and Organism. For the plastid genome assembly, the reads were downloaded through a bash script using the OrganelleSRABuild tool (see below). For the BUSCO nuclear gene reconstruction, the reads were downloaded with Fastq-dump v2.8.1. The mined data are summarized in the table describing the results of the plastome genome assemblies (**Supplementary Table S3**).

### 2.4 Plastid genome reference-based assembly and phylogenetic analysis

Two different methodologies were evaluated for the plastid genome assembly (see section 3.1. for the comparison of both methodologies). The *de-novo* plastid genome assembly was performed with the tool GetOrganelle v1.7.6.1 (Jin et al., 2020). See the reference-based approach (OrganelleSRABuild pipeline) in the next paragraph for more details. Both methodologies were compared by an alignment derived from a Smith-Waterman algorithm with the reference sequence using BlastN v2.12.0 (Camacho et al., 2009). They were also compared mapping the reads used in the assembly using BWA v0.7.17-r1188, and calling variants using FreeBayes v0.9.20-17-g5f1bc44-dirty (Garrison and Marth, 2012). Homozygous and heterozygous variants were counted with the following Linux command line: “grep -v “#” <my_variants.vcf> | cut -f8,10 | sed -r ‘s/.+=//’ | sed -r ‘s/:.+//’ | sort | uniq -c”.

The plastid genome assembly was performed using a reference-based approach. Currently, the following *Nicotiana* species plastome sequences are available at NCBI: *N. attenuata (*PRJNA412786), *N. otophora (*PRJNA359948*)*, *N. sylvestris (*PRJNA257217), *N. tabacum* (PRJNA319578), *N. tomentosiformis (*PRJNA257218), and *N. undulata (*PRJNA74317). Additionally, the *N. benthamiana* plastome was available in-house. Briefly, we mapped the processed reads from publicly available *Nicotiana* sequencing experiments (SRA) against the available and phylogenetically closer *Nicotiana* plastome using Bowtie2 v2.1.0 (Langmead and Salzberg, 2012) and the default parameters. Mapped reads were filtered from the SAM output file using Samtools v1.5-1-g27b628e (Li et al., 2009). Then, the variants were calculated with Freebayes v0.9.20-17-g5f1bc44-dirty (Garrison and Marth, 2012). The VCF file was compressed and indexed with Bgzip v1.3.1 and Tabix v1.3.1. Before the consensus call, the regions of zero coverage were calculated using Bedtools v2.27.1 (Quinlan and Hall, 2010) and the output was filtered with the command “grep -w 0$ my_cov.bed | cut -f1,2,3 > my_cov0.bed.” The consensus sequences were calculated with Bcftools v1.3.1 (Narasimhan et al., 2016) using the command “cat N_benthamiana_Chl_reference.fasta | bcftools consensus -m my_cov0.bed my_variants.vcf.gz | sed -r ‘s/>Niben/>MySpecies/’ > MySpecies_CHLMAP.fasta.” Finally, the consensus sequence was polished using Pilon, for which the following steps were followed: the reads were remapped to the sequence consensus (MySpecies_CHLMAP.fasta) using Bowtie2 v2.1.0 and run on Pilon v1.22 (Walker et al., 2014) using the remapped reads. All these commands were wrapped in a Perl script, OrganelleSRABuild, publicly available from a Github repository (https://github.com/aubombarely/OrganelleSRABuild.)

Plastid genome sequences were aligned with Kalign v2.04 (Lassmann and Sonnhammer, 2005) using default parameters. Two phylogenetic approaches and three different programs were used to build the phylogenetic tree: 1.1) maximum likelihood (ML) using iQTree v2.1.4 (Nguyen et al., 2015) and the substitution model TVM+F+R6 with 1,000 bootstraps. The substitution model was previously generated using jModelTest2 v2.1.7 (Darriba et al., 2012). 1.2) Maximum Likelihood using iQTree v2.1.4. The number of cores was set to AUTO (-nt AUTO), allowing the program to optimize the number of cores for a long alignment. 2) Bayesian inference (BI) using BEAST v2.5.2 (Bouckaert et al., 2019) with the same substitution model as used for 1.1. Phylogenetic trees were generated with FigTree v1.4.0 (Rambaut, 2012).

### 2.5 *De-novo* transcriptome assembly

Reads were downloaded from the NCBI SRA database using Fastq-dump v2.9.6 and then processed with Fastq-mcf v1.04.676 from the Ea-utils package (Aronesty, 2011) (minimum qscore of 30 and minimum length of 50 bp). The processed reads were assembled using Trinity v2.8.5 (Haas et al., 2013) with the default parameters. The transcripts were combined in “supertranscripts” following the recommendations of the Trinity pipeline. The CDS and protein were predicted using Transdecoder v5.5.0 from the Trinity pipeline with the default values.

### 2.6 Reference-based gene model reconstruction

Reads from each of the *Suaveolentes* species were assembled using SOAPdenovo v2.04 (Luo et al., 2012) with kmer sizes of 31, 39, 47, 55, 63, 71, 79, 87, and 95. The assembly was selected based on the longest scaffold, the longest N50, and the largest assembly size. One round of gap filling was performed with GapCloser v2.04 (Luo et al., 2012) (defined as assembly v0.0.5). Scaffolds were broken into contigs again with an in-house Perl script (BreakScaffolds) and re-scaffolded using the *N. benthamiana* reference genome v2.6.1 and RaGOO v1.1. (Alonge et al., 2019) (defined as chimeric assembly v0.1.1). Then another round of gap filling and scaffold break was performed. The final contigs were re-scaffolded with SOAPdenovo v2.04 and a final step of gap filling was run (assembly v0.1.5). The quality of the assemblies was evaluated with BUSCO v4.1.4 (Simão et al., 2015), QUAST v5.0.2 (Gurevich et al., 2013), and Merqury v2020-01-29 (Rhie et al., 2020). Although a basic assembly was performed for all the *Suaveolentes* species (v0.0.5), RaGOO scaffolding was performed only for *N. africana, N. amplexicaulis, N. forsteri* (formerly *N. debneyi*, (Marks, 2010))*, N. cavicola*, and *N. suaveolens* (PI230960) due to the long computing time.

### 2.7 Whole-genome assembly annotation

Whole-genome assemblies were downloaded from the NCBI genome database. For the genomes without available annotations (*N. glauca, N. knightiana, N. obtusifolia, N. paniculata* and *N. undulata*), an RNA-Seq dataset was also downloaded from NCBI and processed as described in the previous section. The processed RNA-Seq reads were mapped to their corresponding references using Hisat2 v2.1.0 (Kim et al., 2015), transcript models were produced with Stringtie v1.3.3 (Pertea et al., 2015), and repeats were annotated with RepeatModeler v2.0.3 (Flynn et al., 2020) (http://www.repeatmasker.org/RepeatModeler/). A set of proteins for the species *S. lycopersicum*, *N. sylvestris*, *N. tomentosiformis*, and *N. attenuata* were downloaded from NCBI and used as closed related protein sets for gene model annotation. From the *Suaveolentes* species, *N. africana*, *N. forsteri*, and *N. cavicola* were annotated with Maker-P v2.31.10 on an Ubuntu server 3.19.0-84 with 256 Gb of RAM, 4 Tb of hard drive, and 64 threads.

### 2.8 Phylogenetic analysis by gene families

All the CDS sequence files were merged with a simple cat command and then gene families were built using program WGD v2018 (Zwaenepoel and Van De Peer, 2019). Specifically, the command “wgd mcl –cds –mcl -s All.CDS.fasta -o All.CDS” was run to create the gene families and then command “wgd ksd –preserve All.CDS.mcl All.CDS.fasta” was run to calculate the Ks distributions. This command kept the intermediate files, such as the alignments produced by MAFFT and the trees produced by Codeml, as part of the WGD script. Different inflation values were assessed (1.5, 2.0, 2.5, 3.0, 3.5, and 4.0) to maximize the number of clusters in which each of the diploid species have one gene and the tetraploid species have two. Alignments with at least one species of the different clades, two *N. benthamiana* sequences and one *S. lycopersicum* sequence, were selected and realigned using Guidance v2.02 (Sela et al., 2015). Then, a first run of iQTree v1.6.12 (Nguyen et al., 2015) was performed on each of the alignments with the default parameters to optimize the substitution model, after which it was run again with the optimal substitution model and 1,000 bootstraps (-bb 1000), a likelihood ratio test (-alrt 1000), and optimization of the UFBoot trees (–bnni). Guidance and iQTree were run for all the alignments using an in-house script called FromAlign2Trees.

### 2.9 Origin and divergence dating of *Suaveolentes*

Identification of the origin and polyploid event dating were performed using the phylogenetic alignments and trees derived from the gene family analysis. The code can be found at the repository: https://github.com/aubombarely/GenoToolBox/tree/master/GeneFamilies In brief, phylogenetic trees were selected based on the following requirements: 1) they should contain at least one sequence of each of the diploid species (*Nicotiana* as well as *Solanum*); 2) they should contain two sequences from *N. benthamiana* and at least another one of each of the other *Suaveolentes* species used to produce the gene families (*N. africana*, *N. forsteri*, and *N. cavicola*); 3) all the nodes of the phylogenetic tree produced by iQTree should have a bootstrap percentage >70. Each of the trees were analyzed using an in-house script called MultiTreeAnalyzer. This script has several functions. First, it tags each of the leaves for polyploid species (*N. africana*, *N. benthamiana*, *N. cavicola*, *N. forsteri*, and *N. tabacum*) with the closest diploid section for which it 1) checks that the different diploid sections are monophyletic, 2) retrieves the common ancestor node for the polyploid leaves and the diploid section, and 3) assigns a tag based on the closest diploid section. If more than one diploid section is under the same node, it will assign multiple origins (e.g., if *Sylvestres* and the *Alatae* clades have a common ancestor and this node is connected with the common recent ancestor of polyploid leaves, it will be assigned as *Sylvestres, Alatae*). Second, the script produces a list of ancestral nodes and the clades under these nodes. Then it counts the number of nodes normalized according to the number of leaves under each node, which represents the frequency for which specific clades are related under a multi-tree analysis.

Once each of the possible polyploid ancestors were identified, each of the gene clusters was classified according to their diploid origin into different types (e.g., Type001 were trees where *Suaveolentes* genes had either *Alatae* or *Noctiflorae* origins) using the ClusterClassification.txt file. Each of the gene family trees were concatenated into a multi-tree file according to their type. An Astral species mapping file was created for each of the types with clear diploid assignment using the PrepareASTRALTaxamap script and Astral v.5.7.8. (Zhang et al., 2018) was run for each of the cluster types with the default parameters. Each of the consensus trees was visualized with Figtree v1.4.4.

Plastid phylogeny dating was performed with BEAST v2.5.2 (Bouckaert et al., 2019), using the split between *Solanum* and *Nicotiana* to calibrate the tree. The gamma site model with a gamma category of 4 and HKY substitution model was selected. The gene tree model was a calibrated Yule Model with previously based on the divergence between the *Nicotiana* and *Solanum* genera. The *Solanum-Nicotiana* split date was estimated at 29.5 Mya based on 21 studies (www.timetree.org). Additionally, gamma distributions with Alpha = 0.001 and Beta = 1,000 were selected for the birth and clock rates. The simulation was run for a chain length of 10 million.

Polyploid event dating using nuclear genes was performed using each of the alignments of the trees classified according to their type. Alignments were converted into FASTA format to change the ID of the sequence using their corresponding species assignment (e.g., Nafr000004 was Nafr_S, Ntom000007 was Ntom) and a simple Perl command. Each of the alignments were changed back to the Phylip format and a BEAST tree phylogeny was estimated for each of the alignments in which the *Suaveolentes* polyploid genes had a clear diploid origin using the same parameters as for the plastid tree. Dating of the different nodes was assigned with Figtree v1.4.4, in which the root node (divergence between *Solanum* and *Nicotiana*) was annotated as 29.5 Mya. Any BA trees that disagreed with the ML trees calculated with iQTree were discarded from the analysis. The *Suaveolentes* ancestor and diploid relative divergence date was calculated as an average of the dating for the different nodes.

An alternative dating was performed based on the Ks distribution obtained from the WGD program (see previous section). The Ks tables were uploaded into R v4.1.2 and the distribution was modeled using function kde() from the package Ks v1.13.5 (Chacón and Duong, 2018). Peaks were found with the function which.max() for the standard R package. The phylogenetic tree produced from the Ks peaks between the different species was produced with the function upgma() from the package Phangorn v2.8.1 (Schliep, 2011) after the KS peak matrix was transformed into a distance matrix using the function as.dist() from the standard R toolbox.

## 3 Results

### 3.1 Plastid genome reconstruction pipeline.

**Figure 1:**
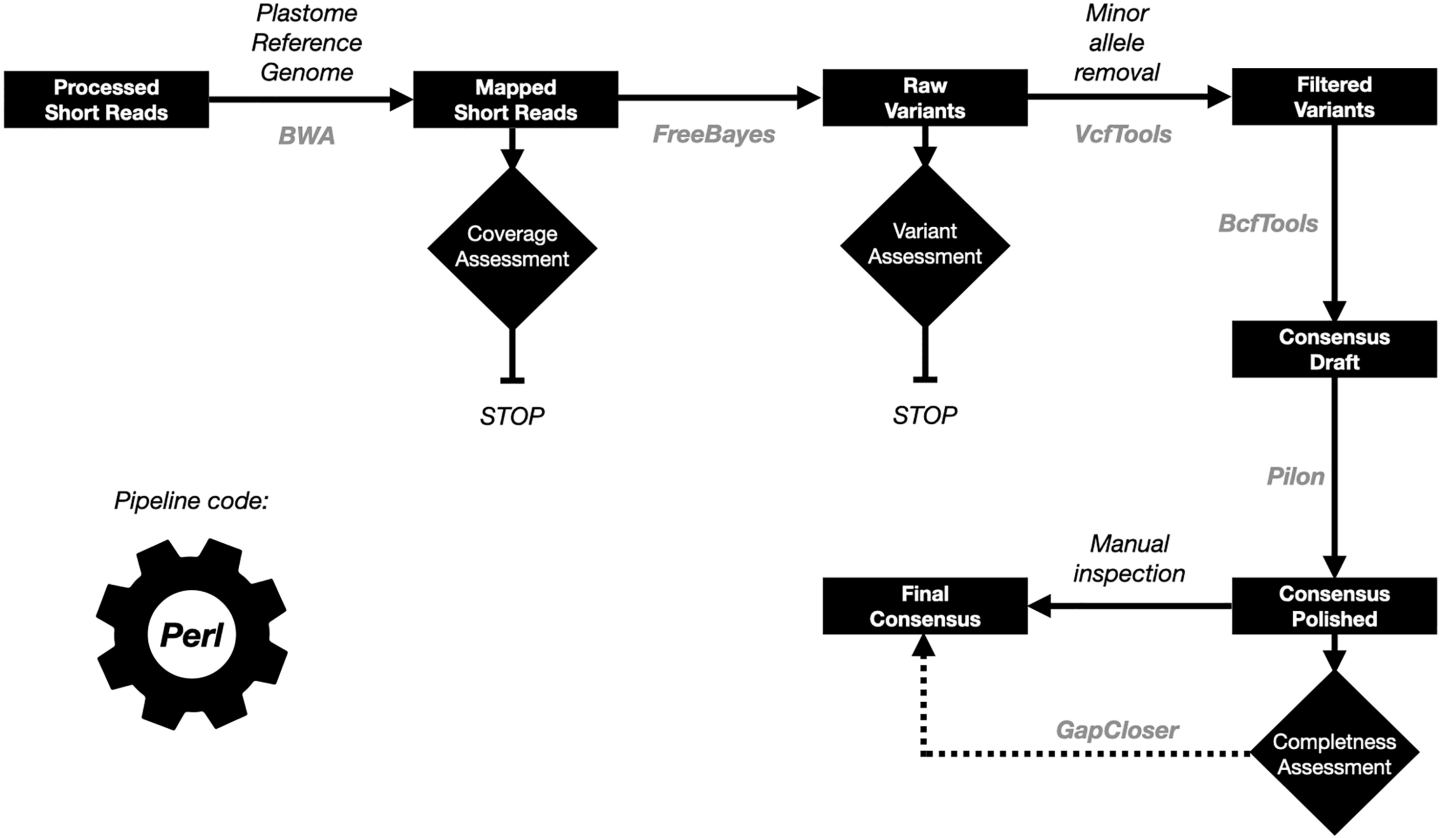
Summary of the plastid genome assembly pipeline. The data is represented in black boxes. The control processes as diamonds. The tools names used in each of the steps are in gray color.

At the time of the publication of this article there were several pipelines to reconstruct plastid genome sequences using short read data, of which GetOrganelle is the most popular one (Jin et al., 2020). We assessed this tool on our whole genome sequencing (WGS) and RNA-Seq data (**Supplementary Table S3**). Although, in general we obtained good results for the WGS data for the datasets with enough coverage (> 20X for the plastid genome), this pipeline failed for all the RNA-Seq data. Due to these results, we decided to develop a reference-guided plastid genome assembly approach called OrganelleSRABuild, in which the reads are aligned with a plastid reference genome and then, variants are called and used to produce a new consensus (see material and methods for more details about the pipeline implementation). The pipeline is summarized in the **Figure 1**.

The OrganelleSRABuild (OSB) pipeline was compared with the GetOrganelle (GO) to assess the accuracy in the plastome genome reconstruction. We assembled the *Arabidopsis thaliana* and the *Nicotiana benthamiana* and *N. africana* plastome genomes. The accuracy was evaluated through the number of homozygous variants identified after the remapping of the assembled reads. More accurate assemblies will have less homozygous variants. We considered that heterozygous variants were produced by NUPTs, especially if the alternative alleles were supported by less than 1/10 of the reads supporting the reference allele. The results are summarized in the **Supplementary Table S4**. Both pipelines delivered the same assemblies for the *A. thaliana* plastome genomes. For the *N. benthamiana* plastome genome assemblies, the GO pipeline produced better results with only one variant (one SNP) compared with the 15 variants (of which five were SNPs) obtained by the OSB pipeline. The use of a reference plastome genome of a species close to our target species was evaluated through the *N. africana* plastome genome assembly using the *N. benthamiana* plastome as reference. Both tools delivered similar results (eight homozygous variants for GO and 10 for OSB), although the number of homozygous SNPs were lower in the OSB assembly (seven compared with the eight of the GO assembly). Considering that insertion/deletion variants produce gaps for the multisequence alignment and that those are ignored for the phylogenetic methodologies used in this work, we decided that the OSB tool produce assemblies better tuned for a phylogenetic analysis. Additionally, GetOrganelle failed to reconstruct any of the plastome genomes derived from RNA-Seq data.

### 3.2 Plastid genome phylogenetic tree present different topologies for the different structural regions

We assessed maternal inheritance of the *Suaveolentes* section through a comparison of the whole-plastome sequences of diploid *Nicotiana* species from all sections. Whole-genome sequencing was performed for 18 *Suaveolentes* species (**Supplementary Table S2**). Plastome genome sequences were obtained and/or reconstructed for 62 taxa (including one outgroup, *Solanum lycopersicum*, and several accessions for three species: *N. amplexicaulis*, two *N. suaveolens*, three; *N. forsteri*, two; *N. glauca*, two; *N. alata*, two; and *N. stocktonii*, two), representing members of all *Nicotiana* clades. Plastome re-assembly was performed for 47 species using the in-house tool OrganelleSRABuild. All assembled plastomes were approximately 0.15 Mb in size. The plastome sequences were assembled without any gap except for *N. petunioides* (1 nt, position 155,690) and *N. raimondii* (1 nt, position 436), (**Supplementary Table S3**). None of the small gaps were in the junction between the IR and the LSC/SSC regions, so they may represent a structural variation in which the polishing methodology may have failed.

**Figure 2:**
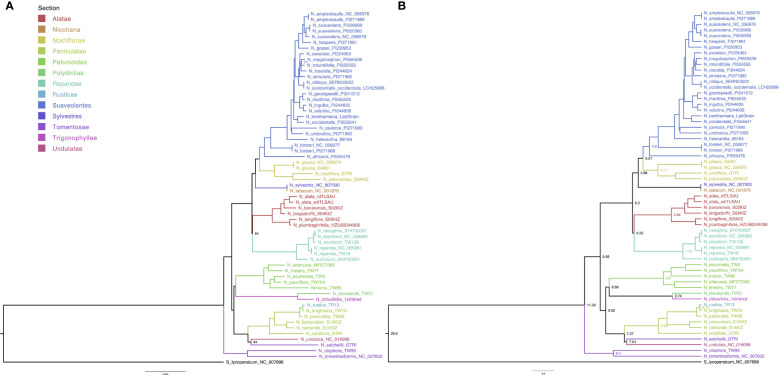
Maternal inheritance analysis of *Nicotiana* section *Suaveolentes* based on whole plastome genome sequence phylogenetic analysis. **(A)** Consensus tree obtained from the maximum likelihood (ML) analysis; B < 70 is indicated in the branch. **(B)** Consensus tree obtained from Bayesian inference (BI) analysis; PP > 0.9; scale bar depicts the years in Million years ago (Mya). Divergence time of the *Suaveolentes* section from the sister clade *Sylvestres* is depicted in a light-blue circle (Mya). Splitting times in Mya between selected sister clades (*Petunioides*/*Trygonophyllae*, *Sylvestres*/*Nicotiana*, and *Alatae*/*Noctiflorae*) are indicated at the bottom in black numbers (10, 5.5, and 0.15). Species are color-coded based on taxonomy (Section), as indicated in the legend. *Solanum lycopersicum* (black) was used as an outgroup. Different species accessions are tagged with accession numbers (**Supplementary Table S3**). Where relevant, the subspecies (subsp.) is indicated.

The whole-plastome alignment was comprised of 62 taxa with 161,044 columns, 4,311 distinct patterns, 3,813 parsimony-informative-, 5,104 singleton-, and 151,927 constant sites. Topology of the phylogenetic tree of the *Nicotiana* genus was independently assessed by ML and BI (**Figure 2**). Both approaches confirmed the previously reported monophyletic nature of the *Suaveolentes* section, with *N. africana* as the oldest ancestor. The ML analysis revealed that most branches were well-supported (B > 90), with only three nodes with B < 90: the *N. megalosiphon* and *N. rotundifolia* ancestral node in the Suaveolentes clade (77); the *N. undulata* and *N. setchellii* ancestral node (84); and the ancestral node for the *Repandae* clade closely nested with the *Alatae* section (84) (**Figure 2A**). BEAST-based BA calculated high posterior probabilities (PP > 0.9) for all nodes (**Figure 2B**). Both topologies revealed *Noctiflorae* as the closest diploid section sister to the *Suaveolentes* section.

Both analyses produced the same topology. To compare our results with a previously published phylogeny, we selected the markers *trnL-F*, *matK*, *trnS-G*, and *ndhF* for a new ML-based phylogenetic analysis (4,260 nucleotides and 124 parsimony informative sites). Our results were like those of previous studies (**Supplementary Figure S1**). In previous analysis in which we used 54 taxa, we obtained topologies in which the *Sylvestres* clade was sister to the *Suaveolentes* section (data no shown). Intrigued by a possible *Sylvestres* phylogenetic signal, we divided the chloroplast genome alignment into three major structural regions: large single copy (LSC), small single copy (SSC), and inverted repeats (IR). Each alignment had 86,293, 25,643, and 21,877 nucleotides with 2,232, 724, and 207 parsimony informative sites, respectively. The LSC and SSC regions produced a ML and BA phylogenetic tree topology in which the *Suaveolentes* and *Noctiflorae* sections were sister groups, whereas the IR region showed similar results to our initial topology, in which *Suaveolentes* and *Sylvestres* were sister groups (**Supplementary Figure S2**). We tested a possible recombination between the *Sylvestres* and the *Noctiflorae* sections on the *Suaveolentes* plastid genomes with the Phi test using two different tools SplitTree (**Supplementary Figure S3**) and PhiPack-Phi. In both cases the phi test did find statistically significant evidence for recombination (p = 1.88E-8 and p = 2.76E-20 respectively) (**Supplementary Table S5**). The significant recombinant regions identified by PhiPack-Phi (p-value < 0.01, length > 5 Kb) were from 89950 to 97700, from 100150 to 111625, from 134075 to 139875, from 140125 to 145525 and from 148000 to 155750 associated with the IR regions.

We estimated the divergence date in the BI dataset (**Figure 2B**) using the *Nicotiana-Solanum* divergence time as calibration in a relaxed-clock model and validated the analysis based on the *N. tabacum* divergence time. Similar to previous results, we dated *N. tabacum* divergence as *circa* 0.06 Mya (Sierro et al., 2014; Edwards et al., 2017). Consistent with previous data (Clarkson et al., 2017; Dodsworth et al., 2020), we estimated the *Suaveolentes* section divergence from the *Sylvestres/Nicotiana* sections to be *circa* 6.34 Mya. We also analyzed the divergence time of the other clades associated with the origins of *Suaveolentes* (**Figure 2B**). While the BI sister clades *Alatae* and *Noctiflorae* split after the divergence of *Suaveolentes circa* 6.50 Mya, the *Petunioides* section branched off with the *Trigonophyllae* section around 9.99 Mya. Our analysis supports *N. knightiana* as the maternal ancestor of *N. rustica* as was reported before (Sierro et al., 2018). Nevertheless our analysis date the polyploidization event as an oldest event (1.48 Mya) compared with the previously published estimates (0.6 Mya (Clarkson et al., 2017)) probably due the use of the whole chloroplast genome, including more variable regions.

### 3.3 *Suaveolentes* nuclear genes have clear contributions from *Alatae, Noctiflorae, Sylvestres* and *Petunioides* sections

To elucidate the complex origin of genome of the members of the *Suaveolentes* section, we performed a phylogenetic analysis of nuclear genes. We selected sequencing data from 19 species representing all the diploid sections of the *Nicotiana* genus, two polyploid sections (*Suaveolentes* and *Nicotiana*), and an outgroup (*S. lycopersicum*) (**Supplementary Table S6**). Our dataset included 4 transcriptome-, 12 genome-, and 3 in-house genome assemblies, including those of *N. africana*, *N. cavicola*, and *N. forsteri*; 9 out 11 DNA whole genome wequence (WGS) datasets were annotated in-house. The genome size of each of the *Suaveolentes* species were estimated by flow cytometry (**Supplementary Table S1**) to estimate the sequencing depth of each of the species. The completeness of the annotations was assessed using the BUSCO set (**Supplementary Table S6**). DNA-based sequencing annotations showed a high degree of completeness. Two species displayed low completeness values (*N. forsteri*, C: 78.1%; *N. otophora*, C: 76%), whereas the rest of the annotations had scores greater than 82.5%. Although some BUSCO values were below the recommended value, we assumed that they were representative of the genome. However, transcriptome-based annotations showed greater variability in completeness (*N. plumbaginifolia*, C: 56.4%; *N. pauciflora*, C: 87.6%), possibly due to the lack of tissue diversity in the datasets. Hence, all datasets were used for further analysis.

**Figure 3:**
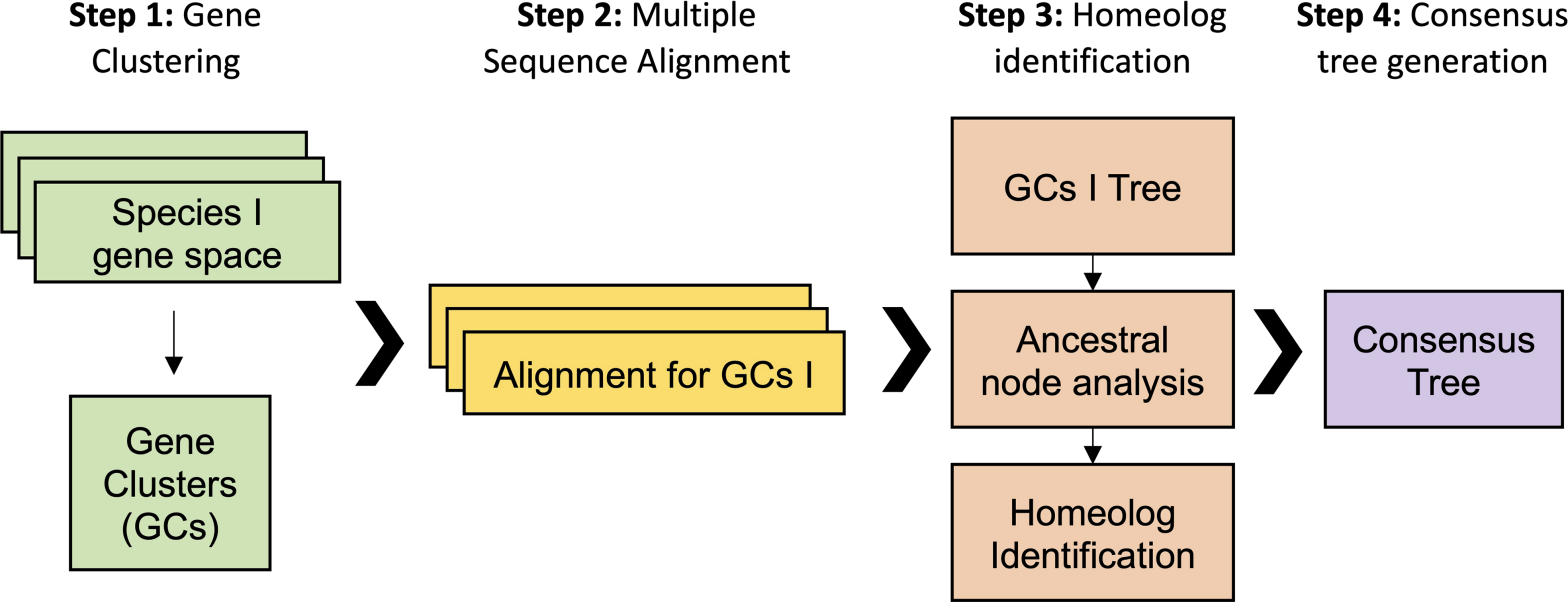
Workflow for the analysis of the origin of homeolog genes in polyploid *Nicotiana* species. The gene family analysis pipeline is divided into four steps. First, gene clustering using protein sequenced. The inflation value (I) was optimized to maximize the number of clusters in which the diploid species have one single copy gene. Second, the mRNA sequence of each gene was aligned for each of the gene clusters. Third, a ML tree was performed for each alignment from which was derived an ancestral node analysis and the homolog identification. Fourth, each of the clusters was classified according its topology and the polyploid homoelog, then a consensus was built for each topology.

Our first analytical approach was based on a gene family analysis (**Figure 3**). First, genes from all previous datasets were clustered in gene families using the Markov cluster algorithm (MCL) (Schaeffer, 2007). In the MCL analysis, the inflation value (I) controls the granularity of the clustering. Thus, variables such as the number of output clusters, number of genes in each cluster, and number of clusters with species-specific genes are controlled by I. We optimized MCL clustering by selecting a I of 2, which displayed the best balance between clusters with single copy genes in diploids and clusters with all the species. We obtained 46,429 clusters, including 8,098 clusters containing all the taxa. The biggest gene family contained 2,346 protein sequences with more than 100 sequences for the *Suaveolentes* species: *N. africana, N. benthamiana, N. cavicola*, and *N. forsteri*. Other *Nicotiana* genomes showed few members (e.g., *N. attenuata*, 8; *N. knightiana*, 1; *N. obtusifolia*, 9; *N. paniculata*, 2; *N. pauciflora*, 1; *N. tabacum*, 59; and *N. undulata*. Second, further analysis revealed that the gene family cluster was a retrotransposon, which was wrongly annotated; the protein encoded by this cluster had the retrotrans_gag_dom (IPR005162) protein domain. Although we did not re-annotate the genomes, the gene family analysis aided in the identification of misannotated repetitive elements. We selected 423 clusters in which diploid species presented one single copy and polyploid species presented at least two copies for *N. benthamiana* and *N. tabacum* and one or two copies for the other *Suaveolentes* species. We used an in-house script to perform the gene family analysis (See Material and Methods). The optimal model in most cases was MGK+F1X4+G4, with or without I. We obtained a total of 411 trees after the filtering (**Supplementary Table S7**).

**Figure 4:**
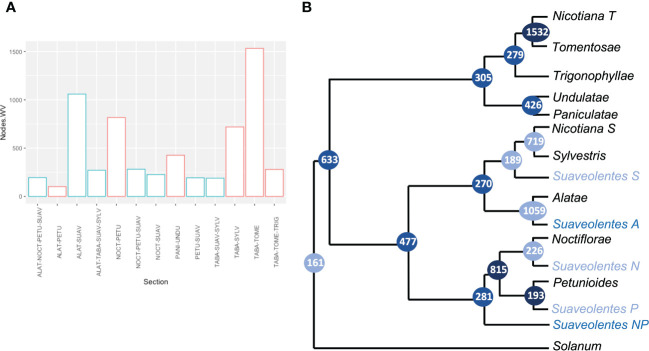
Quantitative analysis of the origin of *Nicotiana* section *Suaveolentes*. **(A)** The number of gene clusters that grouped two, three, or four *Nicotiana* sections together (node weight values). Nodes involving *Suaveolentes* species are colored in light blue. Sections are named as follows: *Alatae* (ALAT), *Petunioides* (PETU), *Suaveolentes* (SUAV), *Noctiflorae* (NOCT), *Nicotiana* (TABA), *Sylvestres* (SYLV), *Tomentosae* (TOME), and *Trigonophyllae* (TRIG). **(B)** Phylogenetic tree diagram based on the gene-family quantification approach. Branch labels represent the number of gene families that cluster in each set of *Nicotiana* sections. Color intensity represents the weight values. Polyploid species homeologs are tagged based on the phylogenetically closer diploid section. P (*Petunioides*), N (*Noctiflorae*), A (*Alatae*), S (*Sylvestres*), and T (*Tomentosae*).

We retrieved each of the nodes to analyze the frequency of the associated sections. As expected, the nodes with a significant weight for the *N.* section (*N. tabacum*) were associated with *N. sylvestris* (719) and *N. tomentosiformis* (1,532) (**Figures 4A, B**; **Supplementary Table S8**, **Supplementary Figure S3**). We also found strong support for *Alatae-Sylvestres* (270) and *Noctiflorae-Petunioides* (815) as diploid sister clades. The number of nodes supporting the structure in which the diploid sections *Alatae-Sylvestres-Noctiflorae* had a common ancestor and the *Petunioides* clade was an outgroup (60) was as probable as the sections *Alatae-Sylvestres-Petunioides* having the same ancestor and the *Noctiflorae* section being the outgroup (77). Similarly, *Undulatae-Paniculatae* had high support as sister diploid clades (426), in agreement with the chloroplast phylogeny. Nevertheless, *Trigonophyllae-Tomentosae* appeared as sister clades (276), instead of *Trigonophyllae-Petunioides* (4). The *Suaveolentes* section showed significant relatedness with *Alatae* (1,059), *Noctiflorae* (226), *Petunioides* (193), and *Sylvestres* (189). The number of nodes in which *Suaveolentes* was a sister group to the ancestor of *Noctiflorae-Petunioides* was also high compared with other nodes (281), which was likely derived from the ancestor of these species instead of a hybridization event between the ancestors of both sections. Nevertheless, the same case was less frequent for the *Alatae-Sylvestres* ancestor (97), compared with the hypothesis in which both sections had independent hybridization events.

**Figure 5:**
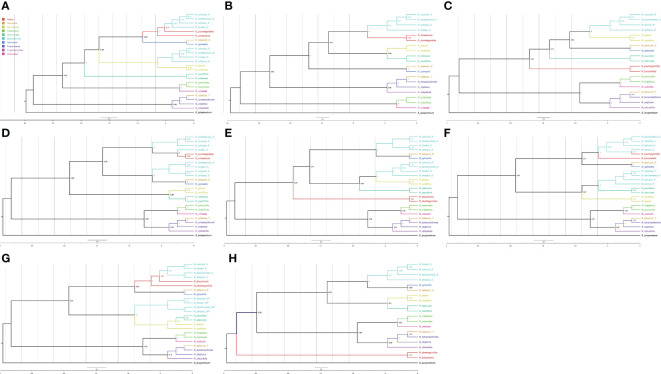
ASTRAL consensus trees for the different ancestral assignments of polyploid leaves. Only the consensus trees produced with more than 10 trees are represented. **(A)** Consensus tree Type001, **(B)** Type002, **(C)** Type003, **(D)** Type004, **(E)** Type005, **(F)** Type006, **(G)** Type007, and **(H)** Type010; suffixes for the *Suaveolentes* leaves are A, *Alatae*; N, *Noctiflorae*; P, *Petunioides*; S, *Sylvestres*; and NP, *Noctiflorae-Petunioides* ancestor. The color for each leaf is indicated in the legend.

Once the tags were assigned to each of the polyploid leaves, we classified 130 types of trees according to the possible ancestors of each polyploid leaf (**Supplementary Table S9**). We discarded 45 trees in which the *N. tabacum* leaves did not have the expected ancestor tags. Of the 130 types, only 31 had 3 or more trees, and these were used for further analysis. We used ASTRAL to calculate the consensus trees for each of the types. The types for which the *Suaveolentes* leaves did not have a clear assignment failed to produce a consensus tree (e.g., Type029). The ASTRAL consensus tree with more phylogenetic trees (Type001, 23 trees), presented a phylogenetic structure in which *Alatae* and *Sylvestres* were sister clades (**Figure 5A**), consistent with the node weight analysis and previously published *Nicotiana* phylogenies, though there were some trees wherein *Noctiflorae* was not a sister clade to the ancestor *Alatae-Sylvestres* (bootstrap 0.58). The contribution of *Alatae* and *Noctiflorae* to the *Suaveolentes* section was well supported (bootstrap > 70); although for *Alatae* derived leaves, *N. forsteri* was the oldest divergent *Suaveolentes* species, and that for *Noctiflorae* was *N. africana*. The second-most frequent topology (Type002, 19 trees) presented only one homeolog for the *Suaveolentes* clade, derived from *Alatae* (**Figure 5B**). *Noctiflorae* and *Petunioides* were well supported sister groups (bootstrap = 0.99). *Alatae* and *Suaveolentes* were also identified as sister groups, though the bootstrap value was low (0.21) compared to that of other topologies with *Alatae* and *Sylvestres* as sister clades. Like Type001, *N. forsteri* was the oldest divergent *Suaveolentes* species in the Type002 topology. The Type003 topology was calculated as a consensus of 16 trees (**Figure 5C**). *Suaveolentes* were represented by one homeolog closely related with the *Noctiflorae* clade. In this topology, *Noctiflorae* and *Sylvestres* were sister clades, and shared the closest ancestor first with *Petunioides* and then with *Alatae* (bootstrap = 0.62 and 0.77, respectively). Like Type001, *N. africana* was the oldest divergent *Suaveolentes*. This was also consistent with the trend in the Type005 consensus tree (**Figure 5E**). However, for the *Alatae* derived *Suaveolentes*, *N. forsteri* usually appeared as an outgroup (**Figures 5A, B, D, F**). Few trees presented Alatae/Sylvestres and the Noctiflorae/Petunioides ancestral nodes as the most close related to the Suaveolentes clade (**Figures 5G**). The ASTRAL consensus tree for *Sylvestres*-derived *Suaveolentes* homeologs had low bootstrap support (**Figures 5D, E, H**). The consensuses trees had good bootstrapping values (>90) (**Supplementary Tables S7**, **S9**), although the topology varied between trees. For example, in tree CL13177, one of the *N. forsteri* homeologs was a sister taxon to *N. africana* under the *Sylvestres* relationship, whereas the other homeolog was associated with the *Alatae* clade. The homeologs of the other *Suaveolentes* taxa were sister groups to the *Sylvestres* clade without *Alatae* derived homeologs. Similar results were observed for CL13194, CL13356, and CL14077, although some bootstrap values were low. Nevertheless, in CL13605 with *Alatae*-derived *Suaveolentes*, *N. africana* was the oldest *Suaveolentes* taxon, whereas that for the *Sylvestres*-derived taxa was *N. forsteri*.

**Figure 6:**
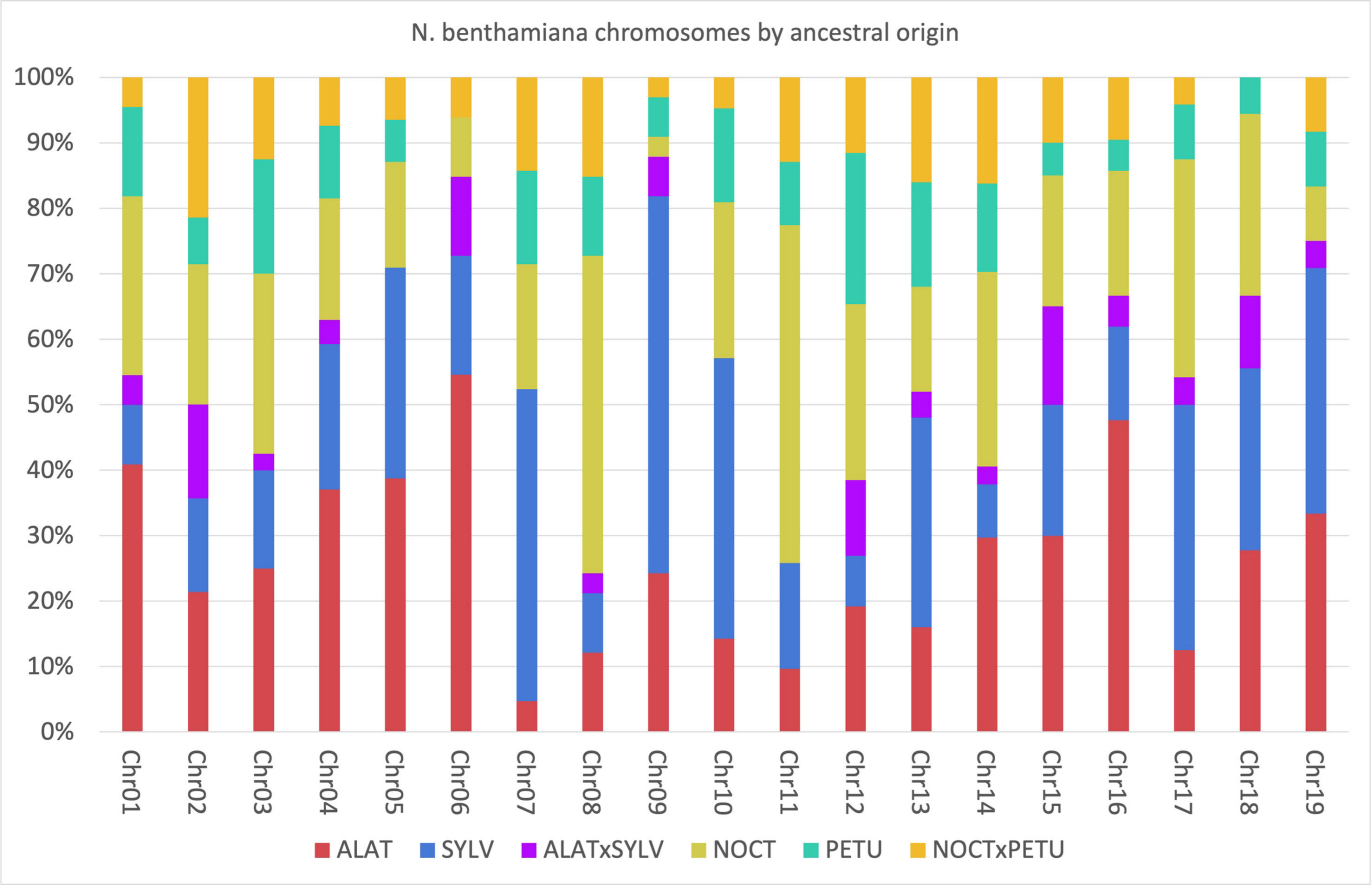
Percentage of the *Nicotiana benthamiana* chromosomes associated to each of the possible origins. Analysis based on the 505 genes for which the origin was identified. Blue, *Sylvestres*; Green, *Petunioides*; Red, *Alatae*; Yellow, *Noctiflorae*; Orange, *Noctiflorae-Petunioide*s ancestor; Purple, *Alatae-Sylvestres* ancestor.

To elucidate the relationship between the different homeologs in the *Suaveolentes* genes, we painted the chromosomes of the *N. benthamiana* reference genome (Niben2.6.0) according to the origin of its homeolog (**Supplementary Figure S5**; **Supplementary Table S10**). Although the chromosomes were a mosaic of different origins, some trends could be observed. Chromosomes were generally enriched in *Alatae* (e.g., 6 and 16; total genome 26.34%), *Sylvestres* (e.g., 7, 9, and 10; total genome 24.36%), and *Noctiflorae* (e.g., 8 and 11; total genome 23.76%) homeologs, with lower contributions from the *Petunioides* homeolog (e.g., 12, total genome 11.48%). Considering enrichments >20% of the homeologs, the most frequent combinations were *Alatae-Sylvestres* (chromosomes 4, 5, 9, 15, and 19), *Alatae-Noctiflorae* (chromosomes 1, 2, 3, and 14), and *Sylvestres-Noctiflorae* (chromosomes 10 and 17). The triple combination *Alatae-Noctiflorae-Sylvestres* was present in two chromosomes (15 and 18) and *Petunioides-Noctiflorae* was present in >20% of the homeologs for chromosome 12 (**Figure 6**; **Supplementary Table S11**).

### 3.4 Molecular dating of the origin of the *Suaveolentes* clade

**Figure 7:**
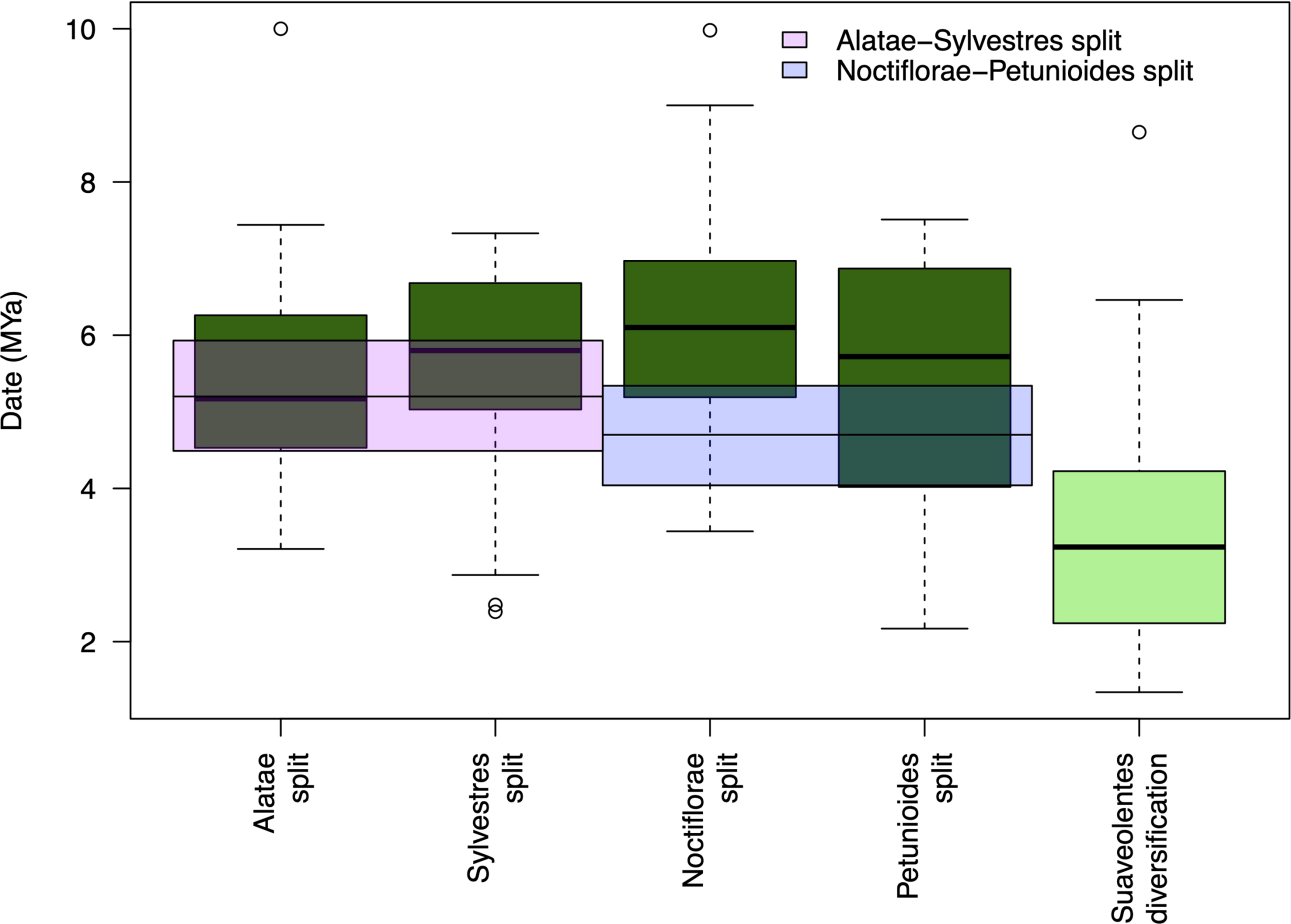
*Suaveolentes* polyploid event dating. The date distribution for the diploid ancestor split with the *Suaveolentes* homeologs as well as the diversification date of the *Suaveolentes* clade calculated from 59 BI trees. The divergency estimate for the diploid species are represented as pink (*Alatae-Sylvestres*) and blue (*Noctiflorae-Petunioides*) boxes.

Our results show different hybridization/polyploidization events that could have generated the *Suaveolentes* section. The split between the *Suaveolentes* homeologs and the different diploid ancestors, *Alatae*, *Noctiflorae*, *Petunioides*, and *Sylvestres*, was dated to 5.37, 6.16, 5.45, and 5,56 Mya, respectively. The diversification event for the *Suaveolentes* section, based on the split of *N. africana*, started around 3.43 Mya (**Figure 7**; **Supplementary Table S12**), which was consistent with chloroplast dating results (5.43 Mya for the split of *Sylvestres-Suaveolentes* and 4.48 Mya for the split of *N. africana* with the rest of the species).

However, it is unclear when the *Alatae, Noctiflorae, Petunioides*, or *Sylvestres* sections diverged and whether this occurred before the Suaveolentes polyploidization event. We estimated the phylogenetic relationship between the diploid accessions and divergence time using a BI tree composed of the 411 gene clusters previously used. This nuclear tree differed in several ways from the whole-chloroplast genome sequence tree. Specifically, *Noctiflorae* was a sister clade to *Petunioides*, whereas *Alatae* was sister to *Sylvestres* (**Supplementary Figure S6**). The divergence date for *Noctiflorae-Petunioides* was 4.70 Mya (height_95%_HPD: 4.04, 5.34) and that for *Alatae-Sylvestres* was 5.20 Mya (height_95%_HPD: 4.49, 5.93), predating the estimated polyploidization event for *Suaveolentes*.

We used a synonymous ratio (Ks) distribution analysis of orthologous genes to estimate the date of the speciation events for the diploid clades and *Suaveolentes* homeologs, as an alternative to the phylogenetic method (Blanc and Wolfe, 2004; Barker et al., 2008; Rabier et al., 2014) (**Supplementary Figure S7**). The split date between the *Alatae* and *Sylvestres* sections (Ks = 0.068) was close to that for *Noctiflorae* and *Petunioides* (Ks = 0.052). Both events were more recent than the divergence between the *N. benthamiana* (Ks = 0.095), *N. africana* (Ks = 0.096), *N. forsteri* (Ks = 0.101), and *N. cavicola* homeologs (Ks = 0.107) as well as the *Solanum-Nicotiana* split (Ks = 0.279) (**Supplementary Figure S8**). The estimated dates are earlier than those estimated with the phylogenetic approach: 7.20, 5.51, 10.02, 10.13, 10.71, 11.39, and 29.5 Mya, respectively (**Supplementary Table S13**). Finally, we constructed a phylogenetic tree with the Ks peak values for the homolog pairs of two representatives of each section (**Supplementary Figure S9**) and dated the nodes according to the *Solanum-Nicotiana* split. Based on the tree, the divergence dates were more recent than in the previous methods; however, the divergence between *Suaveolentes* (5.31 and 5.55 Mya) and its progenitor predated the split between *Alatae* and *Sylvestres* (4.5 Mya) and *Noctiflorae* and *Petunioides* (4.63 Mya).

## 4 Discussion

Our plastid phylogenetic tree results allow us to hypothesize that although *Noctiflorae* is the maternal ancestor, paternal plastid populations from the *Sylvestres* ancestor could have leaked into the newly formed polyploid cells. Then, the recombination of both genomes drove to the evolution of *Suaveolentes* plastid, where the LSC and the SSC are derived from that of the *Noctiflorae* section and the IR from that of the *Sylvestres* section. Although this scenario may appear improbable, the two processes needed for this to occur, i.e., biparental inherence and plastid genome recombination, have previously been described.

Many studies have described heteroplasmic events in angiosperms (McCauley et al., 2005; Ellis et al., 2008; Lambertini, 2016; Ramsey et al., 2019). Potential biparental plastid inheritance (PBPI) has been described in up to 20% of angiosperm genera (Zhang and Sodmergen, 2010). Furthermore, several studies show that *Nicotiana* species present PBPI (Horlow et al., 1990; Svab and Maliga, 2007), including *N. sylvestris* (Thyssen et al., 2012). The hybridization between *N. sylvestris* and *N. undulata* plants showed paternal inheritance for 0.002% of the seedlings, proving that this type of event occurs in modern *Nicotiana* species. Under this scenario, it is reasonable to think that ancestral *Noctiflorae* and *Sylvestres* plastid genomes shared the same polyploid cell in at least a few individuals.

Biparental plastid inheritance is not the only event needed to produce chimeric plastid genomes, recombination is also necessary. Our sequence recombination analysis on the plastid genomes indicates that recombination may occurred during the polyploidization event in the Suaveolentes clade. The application of new generation sequencing techniques, such as long read sequencing, has showed that most plant species have two different chloroplast structures with different orientations of the single copy regions (Wang et al., 2019). The two structures have been interpreted as consequences of a “BIR-like, recombination-dependent replication mechanism between different linear copies of the plastome” (Maréchal and Brisson, 2010), which could explain the recombination between two different chloroplast genomes. Transplastomic *N. tabacum* plants proved that these types of mechanisms are active in *Nicotiana* chloroplasts. After three generations, the deleterious mutations introduced in the chloroplast genome were removed by gene conversions (Khakhlova and Bock, 2006). This is not the only experiment proving plastid genome recombination in *Nicotiana* species. It was already described in *Nicotiana* hybrids between *N. tabacum* and *N. plumbaginifolia* more than 35 years ago (Medgyesy et al., 1985). Although we do not know if the recombined *Noctiflorae-Sylvestres* plastid genomes were favored by natural selection in the recently formed *Suaveolentes* polyploids, previous studies elucidated the evidence of the occurrence of these two events in *Nicotiana* species.

Although we have some evidence about the *Noctiflorae-Sylvestres* plastid genome recombination, these results need to be considered carefully due the technical limitations of our approach. NUclear PlasTid insertions (NUPTs) are difficult to identify with short reads (e.g., 66% of the *Arabidopsis thaliana* NUPTs are longer than 150 bp, (Michalovova et al., 2013)). Our assembly approach assumed that reads derived from the plastid genome will be found in a much higher proportion than reads derived from the nuclear genome (~20% of sequenced reads from leaf tissues are derived from the plastid DNA) (Soorni et al., 2017). As such, the variants derived from NUPTs will be filtered out as minor alleles before a consensus sequence is called on a guided assembly. Nevertheless, a lower proportion of reads derived from the plastid genome may drive to the introduction of variants derived from the nuclear genome producing chimeric assemblies. Long reads are a better approach to detect NUPTs and filter out of the plastid genome assembly but in our case, we only have them for the *N. benthamiana* genome, so we can’t fully discard the introduction of NUPTs variants in the plastid consensus genomes.

Our phylogenomic approach on the Nicotiana nuclear genes identified the following four sections as possible ancestors: *Noctiflorae*, *Petunioides*, *Alatae*, and *Sylvestres*. *Noctiflorae* and *Sylvestres* have been identified as possible ancestors in many previous studies (Aoki and Ito, 2000; Kelly et al., 2013; Clarkson et al., 2017; Schiavinato et al., 2020), sometimes with signals for *Alatae* (e.g., ITS marker (Chase et al., 2003; Kelly et al., 2013)) and *Petunioides* (e.g., GS3’ marker (Kelly et al., 2013)) (Summary on **Supplementary Table S14**). Some of our results agree with those previously published, but some also suggest that *Alatae* and *Petunioides* are involved in the formation of *Suaveolentes* genomes, which is in disagreement with the results of most recent studies (Clarkson et al., 2017; Schiavinato et al., 2020). The elucidation of complex phylogenetic histories requires not only the use of hundreds of genes, but also extensive sampling. Although analysis of the *N. benthamiana* genome revealed the origin of the *Suaveolentes* clade (Schiavinato et al., 2020), the same analysis lacked a representative sample size (some diploid sections were not represented). The absence of any *Alatae* representative drives the unequivocal conclusion of both *Sylvestres* and *Noctiflorae* being the ancestor. In our study, *Alatae* had the same weight as *Sylvestres*, and although not many gene families supported *Petunioides* as an ancestor, there was enough evidence to consider that this section contributed to the origin of the *Suaveolentes* clade, as previously proposed (Kelly et al., 2013; Clarkson et al., 2017). Under this scenario, two hypotheses exist. One, *Suaveolentes* was derived from the hybridization of two hybrids: *Noctiflorae-Petunioides* (with a higher proportion of the maternal genome from *Noctiflorae*) and *Alatae-Sylvestres* (with equal proportions of both). Two, hybridization occurred between the ancestors of each of the two clades (*Noctiflorae-Petunioides* and *Alatae-Sylvestres*) before they split into the modern sections. There is also a possibility in which one hybrid (*Alatae-Sylvestres*) hybridized with the ancestor of the two other sections (*Noctiflorae-Petunioides*).

We used two different molecular dating approaches. We dated each of the speciation events using a multi-coalescent species model (MSCM) with BEAST (Bouckaert et al., 2019) after identifying the possible homeologs in the tree topology. Alternatively, we analyzed the Ks distribution between different gene pairs for each pair of genomes (Blanc and Wolfe, 2004; Zwaenepoel and Van De Peer, 2019). Both approaches have their strengths and weaknesses, though their inherent differences could provide complementary perspectives. With both approaches, the date of the split between *Suaveolentes* homeologs predates the *Noctiflorae-Petunioides* split (**Figure 7**, **Supplementary Figures S8**, **S9**; **Supplementary Tables S12**, **S13**). The split of the *Suaveolentes* homeologs overlapped with the *Alatae-Sylvestres* split determined in the phylogenetic approach, though it also predates this split according to the Ks approach. Considering that two rounds of hybridizations in less than 1 Mya is more unlikely than just between two ancestors before they split into the major sections, we propose that the *Suaveolentes* section originated from the hybridization of the *Noctiflorae-Petunioides* and *Alatae-Sylvestres* ancestors before their split into these sections.

The divergence date between the *Suaveolentes* homeologs and diploid species derived from the *Suaveolentes* ancestors (5.37 to 6.36 Mya) estimated by a calibrated MSCM tree agreed with previously published results (Clarkson et al., 2017; Schiavinato et al., 2021). Unfortunately, the absence of fossil records limits the possibility of validating our results using a more accurate type of evidence for the dating for which we used an alternative molecular dating methodology. Our estimations based on Ks distributions between homologs generated earlier dates than those obtained by the calibrated MSCM phylogenetic tree (**Supplementary Figures S6**, **S8**, **S9**, **Supplementary Table S13**). The dating differences in both methodologies are greater between the *Suaveolentes* homeologs (~10 Mya compared with 5.37 to 6.36 Mya) than the diploid divergence dates (5.51 and 7.20 Mya for the split in *Noctiflorae-Petunioides* and *Alatae-Sylvestres*, respectively). Although we could attribute some of these differences to an incomplete assembly in the *N. africana*, *N. forsteri*, and *N. cavicola* genomes (**Supplementary Table S6**), our *N. benthamiana* genome delivered similar results with a comparatively complete assembly (C: 97.7% by BUSCO, C: 98.13% by Merqury). Dating whole-genome duplication events by homolog Ks distribution has some important limitations, though older events are generally less accurate than those considered in this work (Vanneste et al., 2013). Nevertheless, it has been proposed that the ratio of synonymous substitutions is higher between homeologs in a polyploid than two diploid species in the overestimation of WGD event dating (Vanneste et al., 2013). We have also to consider alternative scenarios in which the WGD event dating has been underestimated by the molecular dating methodologies. Our dates assume that the *Suaveolentes* dispersal from South America to Australia was produced through long distances by wind *via* the Atlantic and Indian oceans (Clarkson et al., 2004), and the Pacific ocean (Clarkson et al., 2010). Nevertheless, it also has been proposed that the *Suaveolentes* species arrived to Australia through an Antarctic land bridge that connected South America and Australia (Goodspeed, 1954). Based on the disjunct distribution of many related Australian and South American taxa, it has been proposed that this Antarctic land bridge existed at least until 30 Mya (van den Ende et al., 2017) which could push the origin of the *Suaveolentes* clade to the Oligocene.

## Data availability statement

The datasets presented in this study can be found in online repositories. The names of the repository/repositories and accession number(s) can be found below: https://www.ncbi.nlm.nih.gov/, PRJNA853913.

## Author contributions

LD’A performed the chloroplast phylogenetic analysis and article writing. SO performed the DNA extraction and sequencing. NS participated to the DNA extraction and sequencing and on the article writing and project design. TH and ER performed initial experiments associated to this study and participated in the experimental design. NI participated on the article writing and project design. AB performed the genome assemblies and annotations, nuclear phylogenies, script writing, article writing and project design. All authors contributed to the article and approved the submitted version.
