# Supplementary material for: Polyploid Nicotiana section Suaveolentes originated by hybridization of two ancestral Nicotiana clades
Source: Front Plant Sci. 2023 May 8;14:999887. doi: 10.3389/fpls.2023.999887 (PMC10200995; doi:10.3389/fpls.2023.999887)
Supplement: Supplementary file 1 [file DataSheet_1.pdf]

SUPPLEMENTAL

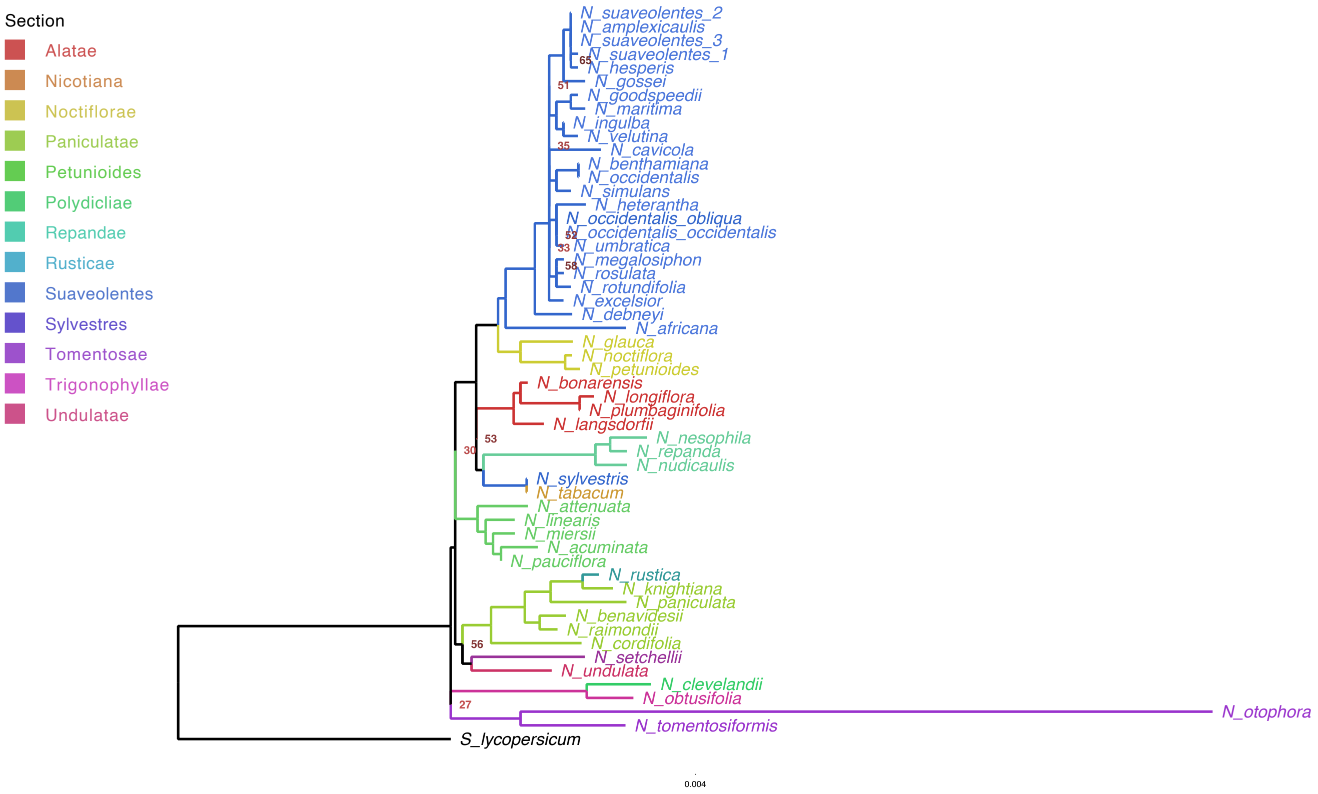

Figure S1. Phylogenetic tree for the plastid markers trnL-F; matK; trnS-G; ndhF using a Maximum Likelihood approach. The bootstrapping values under 70 are represented with red font.

SUPPLEMENTAL

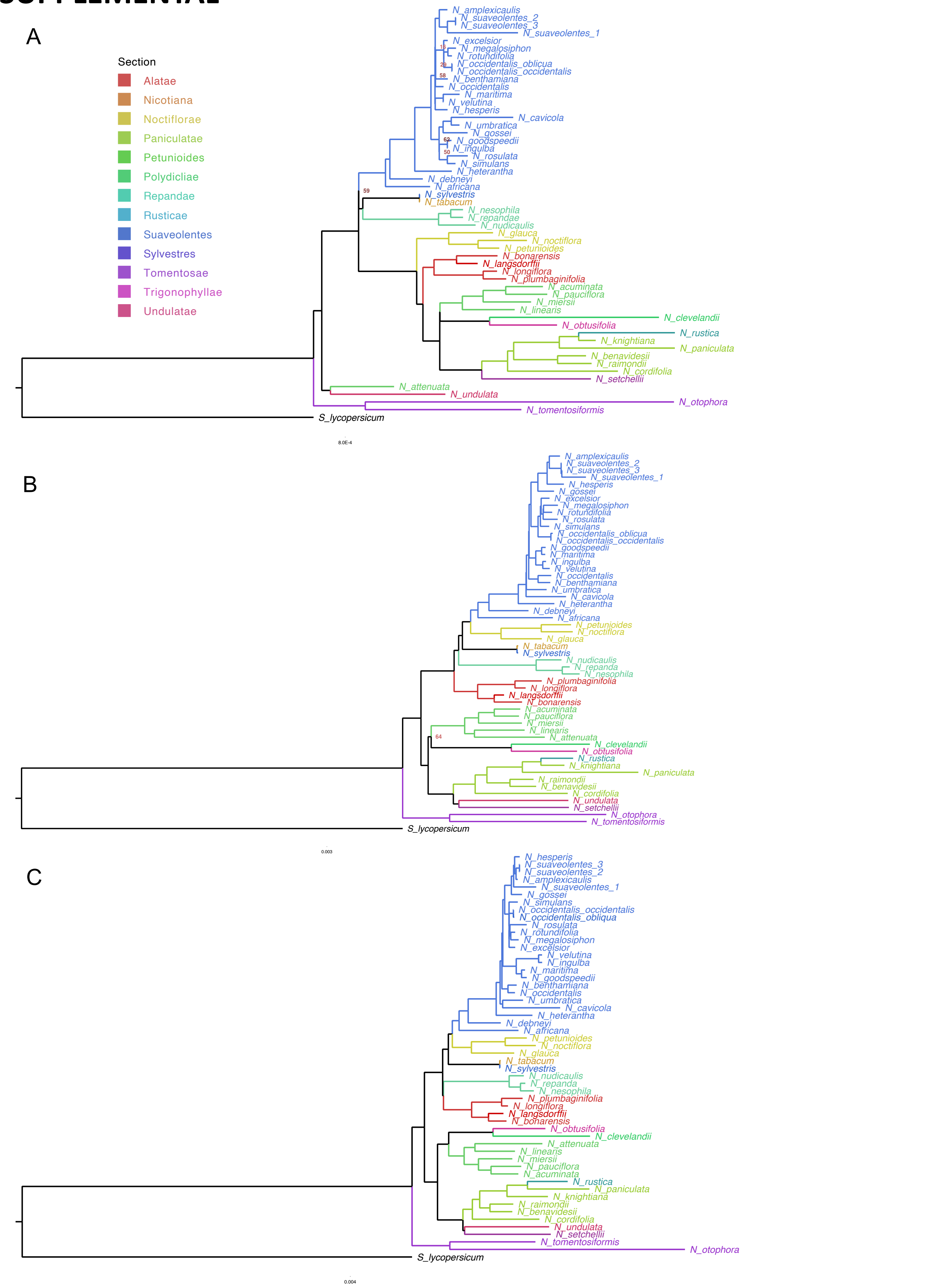

Figure S2. Phylogenetic tree for the plastid regions using a Maximum Likelihood approach. The bootstrapping values under 70 are represented with red font. (A) Inverted Repeat (IR) region; (B) Long Single Copy (LSC) and (C) Short Single Copy (SSC)

SUPPLEMENTAL

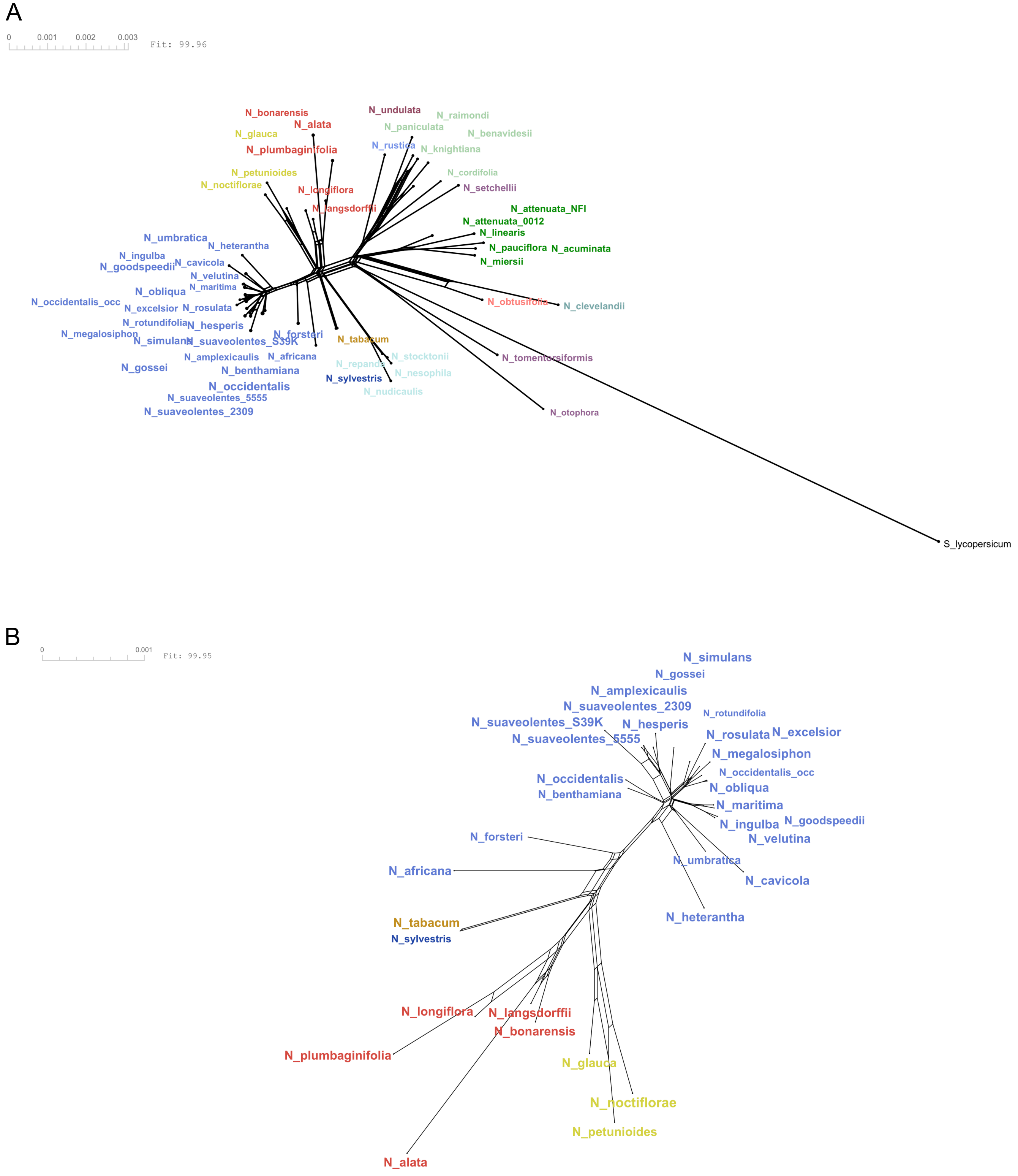

Figure S3: Phylogenetic network produced by Splitstree. Sections are colored as follows: *Alatae* (red), *Nicotiana* (brown), *Noctiflorae* (yellow), *Paniculatae* (light green), *Petunioides* (green), *Polydicliae* (blue-green), *Repandae* (light blue), *Rusticae* (pale blue), *Suaveolentes* (blue), *Sylvestres* (navy blue), *Tomentosae* (purple), *Trigonophyllae* (pale red) and *Undulatae* (dark brown). (A) All the Nicotiana sections; (B) *Suaveolentes, Alatae, Noctiflorae, Sylvestres/Nicotiana* sections only.

SUPPLEMENTAL

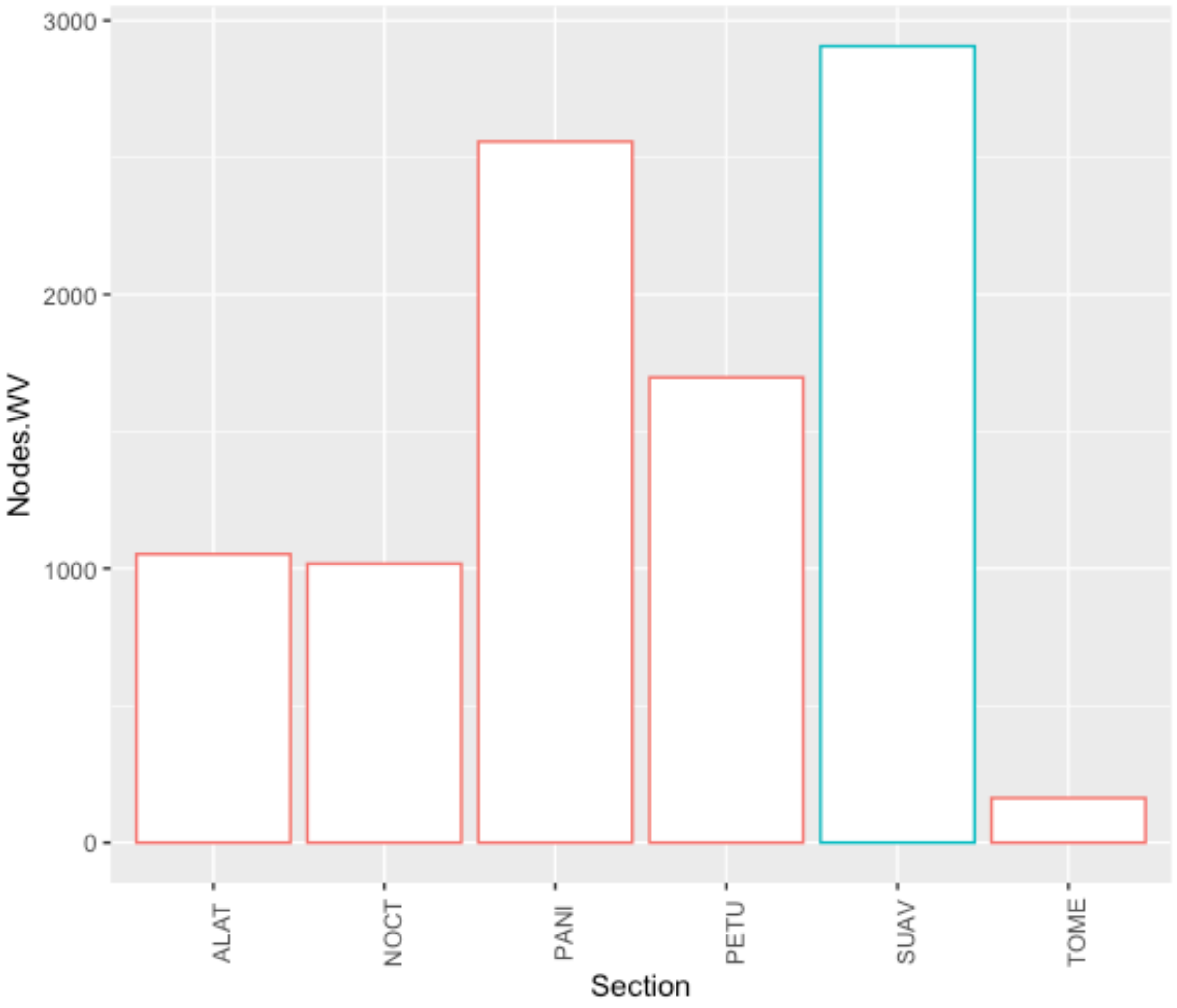

Figure S4. Quantitative analysis of the Nicotiana section monophyletic nature. Histogram plot despict the number of gene clusters that grouped species from a single Nicotiana section together (nodes weights values). Bar representing Suaveolentes section is colored in light blue, while the rest is red. Sections are named as followed: Alatae (ALAT), Petunoides (PETU), Suaveolentes (SUAV), Noctiflorae (NOCT), Tabacum (TABA), Sylvestris (SYLV), Tomentosae (TOME), and Trigonophyllae (TRIG).

SUPPLEMENTAL

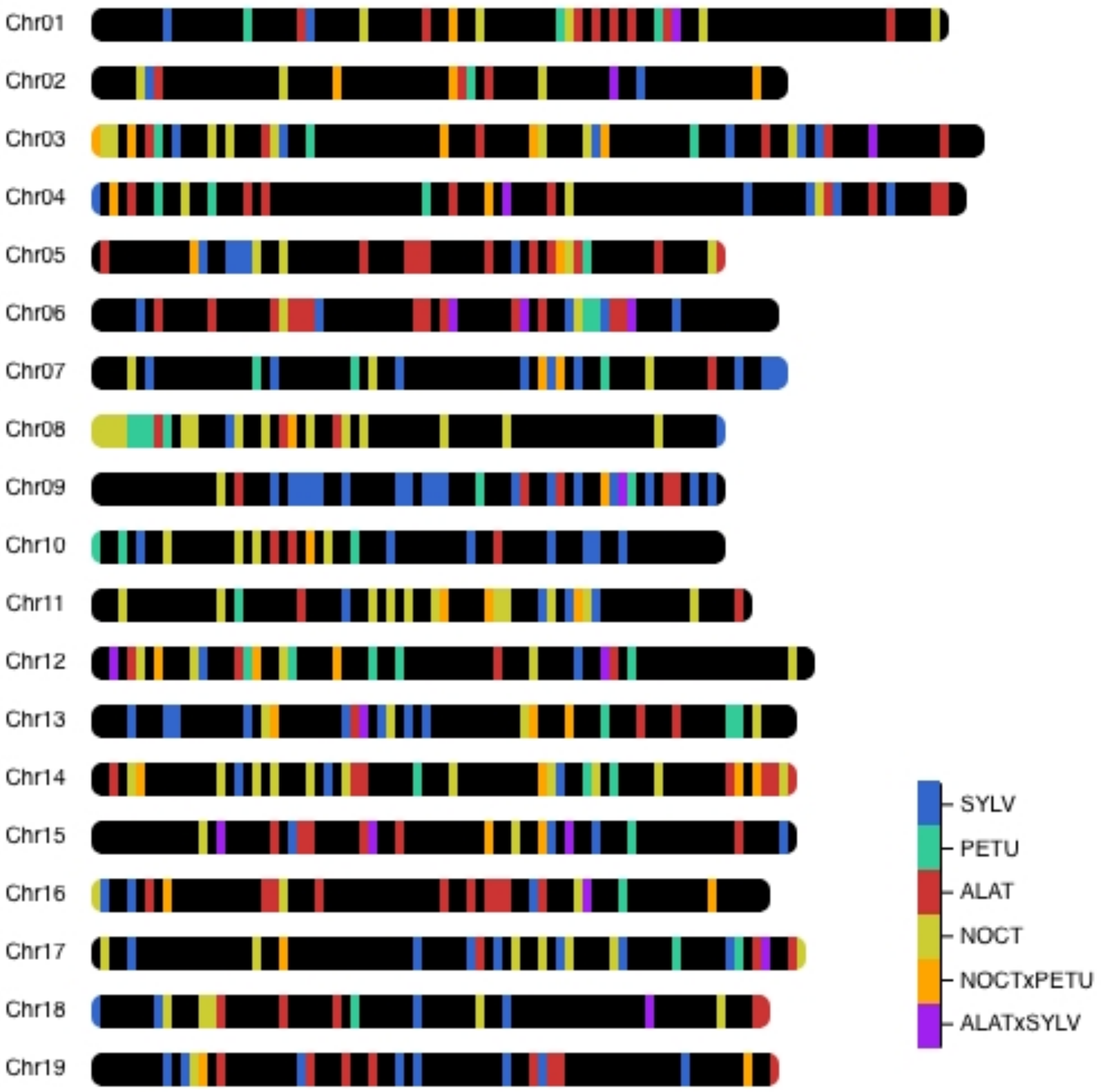

Figure S5. Representation of the origin of Nicotiana benthamiana chromosomes. Painting of 505 genes for which the origin was identified. Blue, Sylvestres; Green, Petunioides; Red, Alatae; Yellow, Noctiflorae; Orange, Noctiflorae-Petunioides ancestor; Purple, Alatae-Sylvestres ancestor.

SUPPLEMENTAL

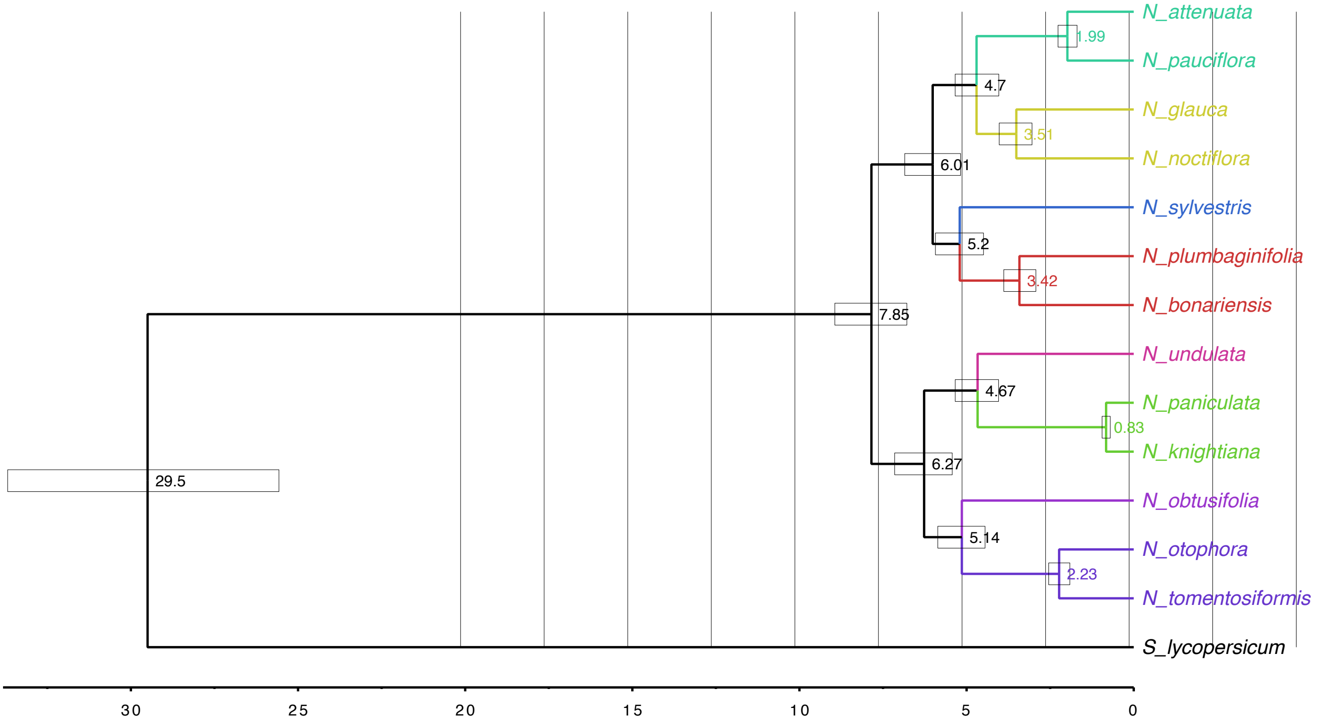

Figure S6. Bayesian phylogenetic tree of the *Nicotiana* taxa representing each of the diploid sections. Colors represent the different sections: aquamarine for *Petunioides*, yellow for *Noctiflorae*, blue for *Sylvestres*, red for *Alatae*, pink for *Undulatae*, green for *Paniculatae*, purple for *Trigonophyllae* and violet for *Tomentosae*. The divergence date is indicated in each of the nodes. The blue node bars represent height 95% HPD.

SUPPLEMENTAL

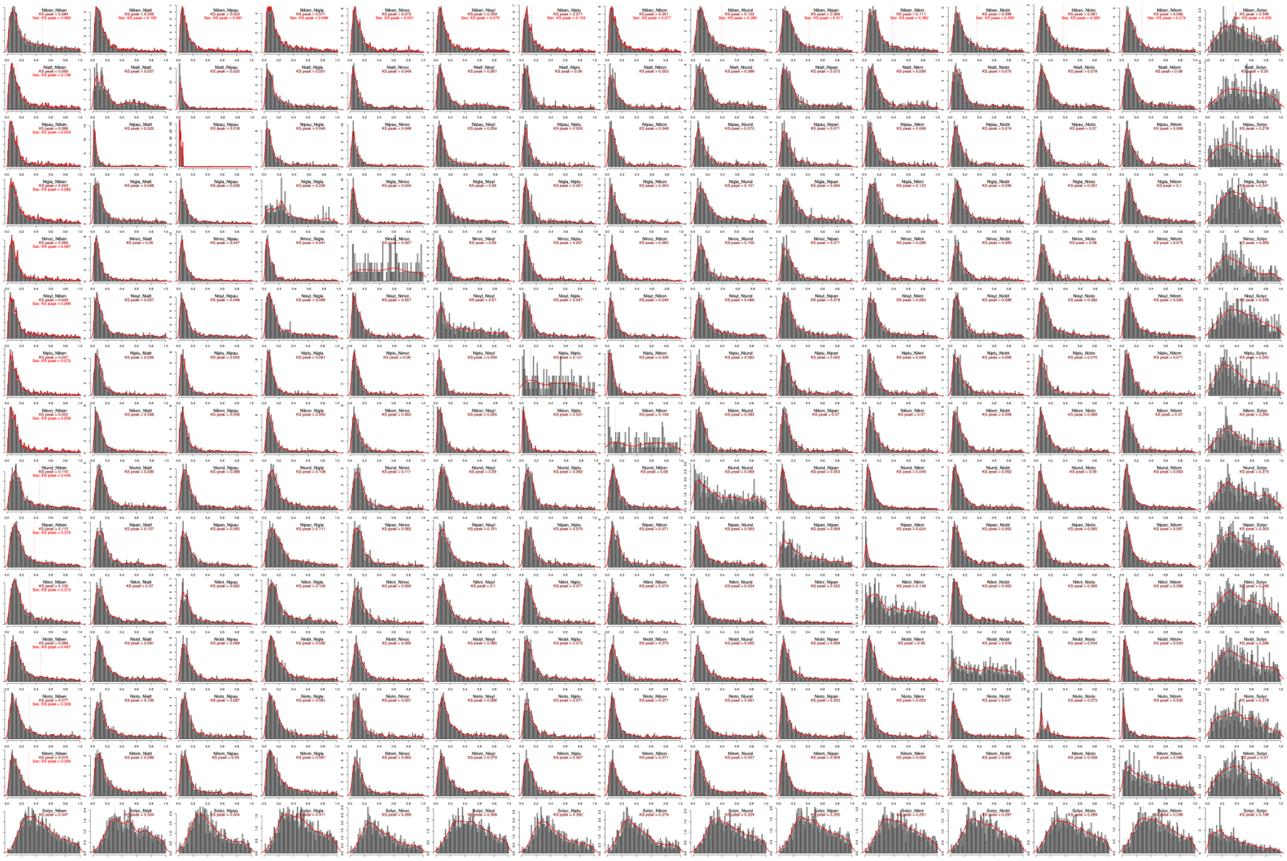

Figure S7. KS distribution of the homolog gene pairs for *Nicotiana* species using *S. lycopersicum* as outgroup. A panel of 15x15 species pair is presented in the following species order: *N. benthamiana* (Niben), *N. attenuata* (Niatt), *N. pauciflora* (Nipau), *N. glauca* (Nigla), *N. noctiflora* (Ninoc), *N. sylvestris* (Nisyl), *N. plumbaginifolia* (Niplu), *N. bonarensis* (Nibon), *N. undulata* (Niund), *N. paniculata* (Nipan), *N. knightiana* (Nikni), *N. obtusifolia* (Niobt), *N. otophora* (Nioto), *N. tomentosiformis* (Nitom) and *S. lycopersicum* (Solyc). The main peak detected in the Kernell Density Estimation (KDE) distribution is marked with a dark-red line and the KS value written with dark-red letters. The secondary peaks are marked with red letters for the *N. benthamiana* distributions.

SUPPLEMENTAL

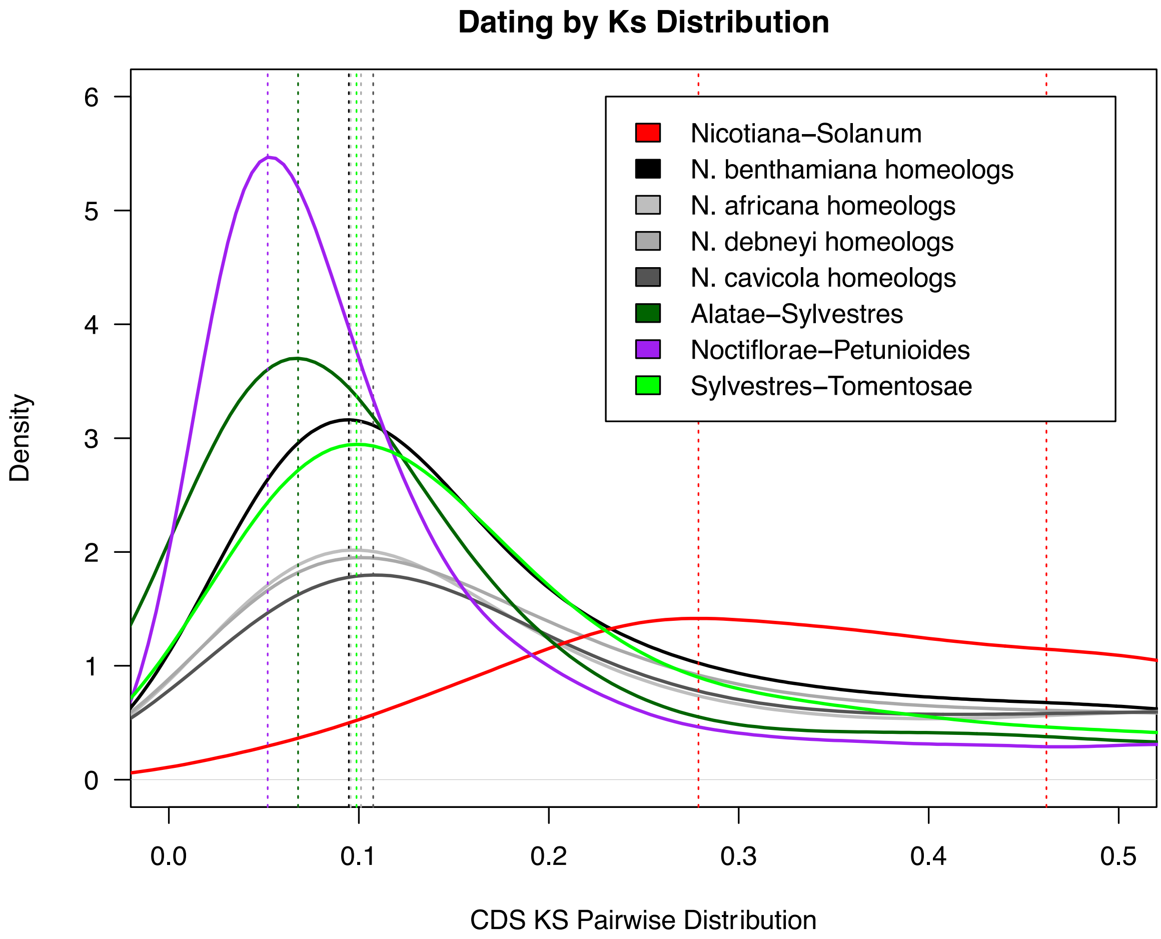

Figure S8. KS distribution for the Nicotiana species. Split *Alatae-Sylvestres*, dark-green (KS=0.068), split *Noctiflorae-Petunioides*, purple (KS=0.052); *Sylvestris-Tomentosiformis*, light-green (KS=0.099), *Solanum-Nicotiana*, red (KS=0.279), *N. benthamiana* homeologs, black (KS=0.095), *N. africana* homeologs, light-gray (KS=0.096), *N. debneyi*, gray (KS=0.101) and *N. cavicola*, dark-gray (KS=0.107).

SUPPLEMENTAL

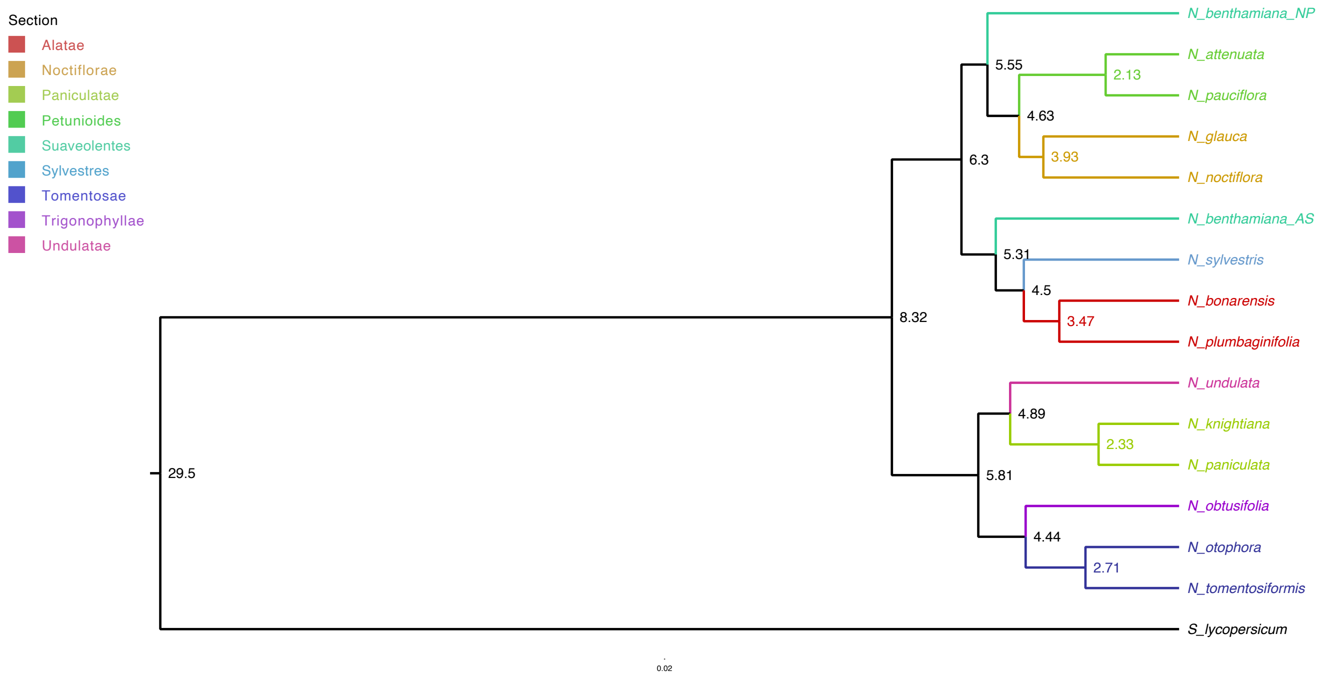

Figure S9. Nicotiana phylogenetic tree built with KS peaks. Species are colored by sections: *Alatae* (red), *Noctiflorae* (yellow), *Paniculatae* (yellow-green), *Petunioides* (green), *Suaveolentes* (blue-green), *Sylvestres* (blue), *Tomentosae* (dark-blue), *Trigonophyllae* (violet) and *Undulatae* (purple). Split dates are indicated in each node.
